# Generation of an external guide sequence library for a reverse genetic screen in *Caenorhabditis elegans*

**DOI:** 10.1186/1472-6750-9-47

**Published:** 2009-05-20

**Authors:** Qitao Yan, Rui Zhao, Wenlin Zheng, Changxin Yin, Bao Zhang, Wenli Ma

**Affiliations:** 1 Institute of Molecular Biology, Southern Medical University, Guangzhou, 510515, PR China; 2 Southern China Genomics Research Center, Guangzhou, 510800, PR China; 3 Gendustry Development Group (GDG), Guangzhou, 510800, PR China

## Abstract

**Background:**

A method for inhibiting the expression of particular genes using external guide sequences (EGSs) has been developed in bacteria, mammalian cells and maize cells.

**Results:**

To examine whether EGS technology can be used to down-regulate gene expression in *Caenorhabditis elegans *(*C. elegans*), we generated EGS-Ngfp-lacZ and EGS-Mtgfp that are targeted against *Ngfp-lacZ *and *Mtgfp *mRNA, respectively. These EGSs were introduced, both separately and together, into the *C. elegans *strain PD4251, which contains *Ngfp-lacZ *and *Mtgfp*. Consequently, the expression levels of *Ngfp-lacZ *and *Mtgfp *were affected by EGS-Ngfp-lacZ and EGS-Mtgfp, respectively. We further generated an EGS library that contains a randomized antisense domain of tRNA-derived EGS ("3/4 EGS"). Examination of the composition of the EGS library showed that there was no obvious bias in the cloning of certain EGSs. A subset of EGSs was randomly chosen for screening in the *C. elegans *strain N2. About 6% of these EGSs induced abnormal phenotypes such as P0 slow postembryonic growth, P0 larval arrest, P0 larval lethality and P0 sterility. Of these, EGS-35 and EGS-83 caused the greatest phenotype changes, and their target mRNAs were identified as ZK858.7 mRNA and *Lin-13 *mRNA, respectively.

**Conclusion:**

EGS technology can be used to down-regulate gene expression in *C. elegans*. The EGS library is a research tool for reverse genetic screening in *C. elegans*. These observations are potentially of great importance to further our understanding and use of *C. elegans *genomics.

## Background

RNase P catalyzes the maturation of 5'-termini of all tRNAs by a single endonucleolytic cleavage of their precursors[1]. This enzyme is found in cells from all three domains of life: the Bacteria, Eukaryote and Archaea [2-5]. One of the unique features of RNase P is its ability to recognize the structures, rather than the sequences, of tRNAs; this allows the enzyme to cleave other substrates with similar structure to the tRNA precursor. Accordingly, any complex of two RNA molecules that resembles a similar tRNA molecule can be recognized and cleaved by RNase P [6-8]. One of the two RNA molecules that resemble the complex is termed the external guide sequence (EGS). In principle, an mRNA sequence can be targeted for RNase P cleavage by hybridization with EGS to direct RNase P to the cleavage site. Subsequent studies have shown that EGS technology can be used to down-regulate gene expression in many organisms, such as bacteria, [9-12] mammalian cells [13-19]and maize cells[20].

Nucleic-acid-based gene-interference strategies, such as anti-sense oligonucleotides, ribozymes, and RNAi, are powerful research tools and promising therapeutic agents for human diseases [21-25]. Each technology has advantages and limitations in terms of targeting efficacy and specificity [26]. Compared with other nucleic-acid-based gene-interference strategies, such as the RNAi approach that induces the cellular RISC RNase to cleave a target mRNA [26,27], targeted cleavage of mRNA by RNase P using an EGS is a unique approach that can be used to inactivate any RNA of known sequence expressed *in vivo*. Moreover two types of interaction govern the targeting specificity of EGS[3,19]. One is the Watson-Crick base-pairing interaction between the anti-sense domain of an EGS and the accessible region of a target mRNA. The other is the interaction between a target mRNA and the other domains of an EGS, which are required for folding of the RNase P-recognizable tertiary structure.

Several EGSs derived from natural tRNA sequences have been shown to be effective in blocking gene expression in bacteria[12,28] and mammalian cells[29]. For example, the "3/4 EGS" resembles three-quarters of the tRNA molecule and consists of two sequence elements: a targeting sequence that is complementary to the accessible region of a target mRNA in which most sequences are inaccessible owing to the secondary or tertiary structures of the RNA and or the binding of proteins; and a RNase-P-recognizing sequence that is a portion of the tRNA sequence and required for interacting with RNase P[8]. It has been demonstrated that the "3/4 EGS" effectively and specifically induces target mRNA cleavage by eukaryotic RNase P [8,28].

Phenotype changes have been associated with more than 1,500 *C. elegans *genes through a combination of RNAi screens, classical mutant screens and systematic gene knockout experiments [30-42]. Despite these successes, the functions of most of the approximately 20,000 predicted genes in the *C. elegans *genome remain elusive. Moreover, there were some clear differences in the results of these RNAi screens conducted by different researchers. These differences were considered to result from different approaches and standards in RNAi screening. Furthermore, there was also 10 to 30% variability in the results of the RNAi screens conducted by the same researcher according to the same procedure [30,31,34,36-40,43,44]. The relative variability of the RNAi effect should be an important consideration before the RNAi data are used as starting point for new experiments[40]. In this study, we show that EGS technology can be used to down-regulate gene expression in *C. elegans*, and the EGS library can facilitate a reverse genetic screen similar to that possible with an RNAi library

## Results

### Validation of EGS technology for down-regulating gene expression in *C. elegans*

**Figure 1:**
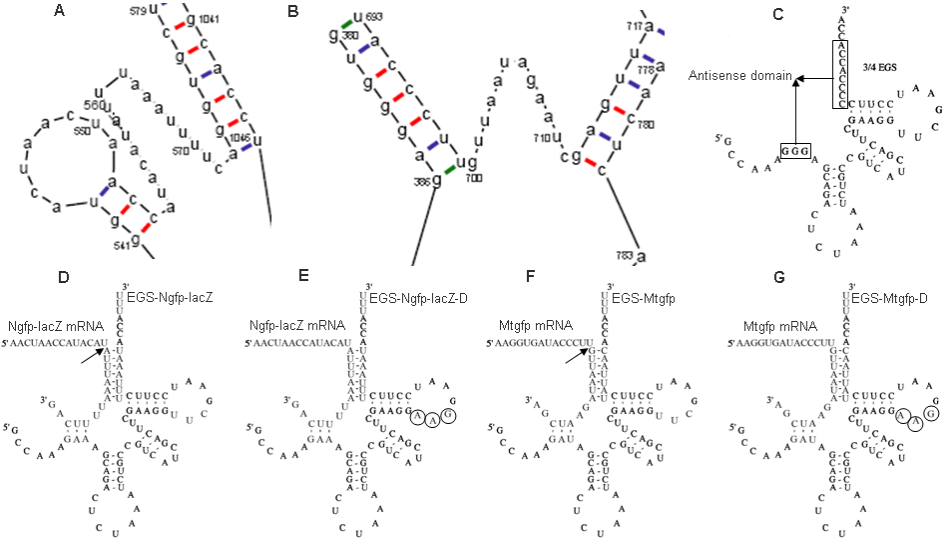
**EGSs targeted to Ngfp-lacZ and Mtgfp mRNA**. (A) The looped region of "auuuaaauuuuc" was chosen as a target region for EGS-Ngfp-lacZ. (B) The looped region of "guuaauagaauc" was chosen as a target region for EGS-Mtgfp. (C) The "3/4 EGS" derived form was a precursor of tRNA^Tyr ^in *Escherichia coli*. (D, E) Complex between *Ngfp-lacZ *mRNA and EGS-Ngfp-lacZ (D) or EGS-Ngfp-lacZ-D (E). The arrow indicates the site of cleavage by RNase P. EGS-Ngfp-lacZ-D is derived from EGS-Ngfp-lacZ by introducing base-substitution mutations at three positions (highlighted by circles) in the T-loop. (F, G) Complexes between the *Mtgfp *mRNA and EGS-Mtgfp or EGS-Mtgfp-D, respectively. The arrow indicates the site of RNase P cleavage. EGS-Mtgfp-D is derived from EGS-Mtgfp by introducing base-substitution mutations at three positions (highlighted by circles) of the T-loop.

There are two types of green fluorescent proteins (GFP) in *C. elegans *strain PD4251. Ngfp consists of a wild-type GFP and a nuclear-localization signal encoded by *Ngfp-lacZ*. Mtgfp consists of a wild-type GFP and a mitochondrial-localization signal encoded by *Mtgfp*[45]. EGSs that target to *Ngfp-lacZ *or *Mtgfp *mRNA can be designed using RNA-folding software[46]. According to the rules of EGS design[28], the favorable accessible regions of *Ngfp-lacZ *(Fig. 1A) and *Mtgfp *mRNAs (Fig. 1B) were identified from all candidate accessible regions. The "3/4 EGS" (Fig. 1C) was used as the framework. The anti-sense sequence of the accessible region was introduced into the antisense domain of the framework. The "CCA" sequence[7,8,28,47,48] located in the 3'-terminus is important for the EGS effect. To protect the "CCA" sequence from being exposed directly to RNase, the "UUU" sequence was attached to its 3'-terminus. Two EGSs, EGS-Ngfp-lacZ (Fig. 1D) and EGS-Mtgfp (Fig. 1F), were constructed. Two additional EGSs, EGS-Ngfp-lacZ-D (Fig. 1E) and EGS-Mtgfp-D (Fig. 1G), were also constructed. EGS-Ngfp-lacZ-D and EGS-Mtgfp-D were derived from EGS-Ngfp-lacZ and EGS-Mtgfp, respectively, and contained point mutations (5'-TTC-3' → AAG) at the three highly conserved positions in the "T-loop" of these EGSs. These nucleotides have been found in most of the known, natural tRNA sequences[49] and are thought to be important for interactions between the tRNA domains and human RNase P[3]. Previous studies have shown that EGSs with these mutations prevented RNase P recognition and showed little activity in directing RNase-P-mediated cleavage[19,50,51].

**Figure 2:**
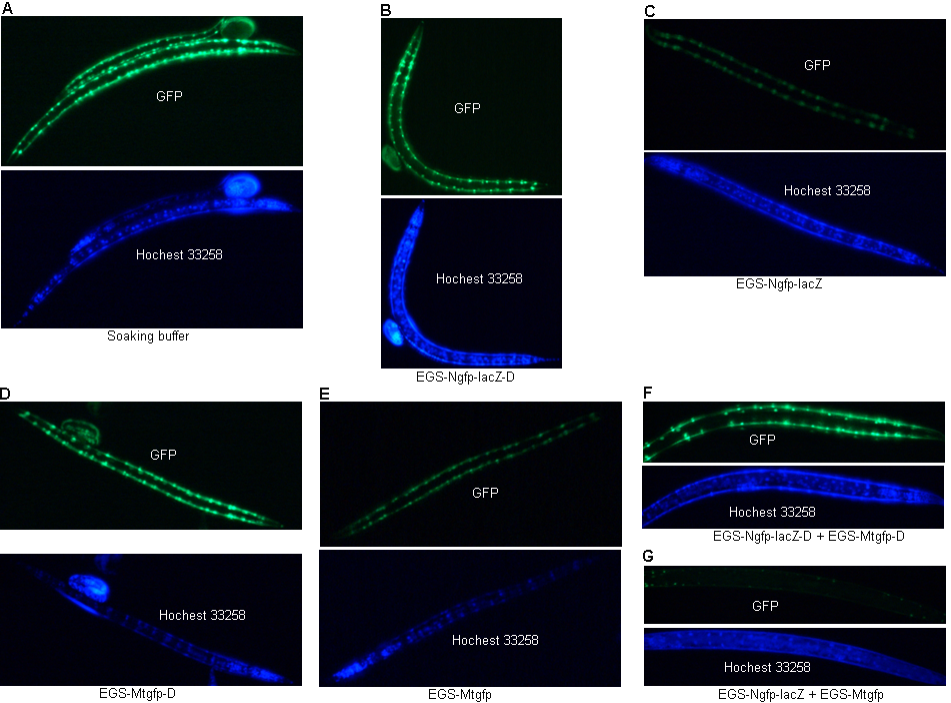
**Effects of EGS on GFP fluorescence of PD4251 worms**. (A) Worms were treated with soaking buffer. (B, D, F) Worms were treated with disable EGSs (EGS-Ngfp-lacZ-D (B), EGS-Mtgfp-D (D), or a mix of EGS-Ngfp-lacZ-D and EGS-Mtgfp-D (F)). (C, E, G) Worms were treated with EGSs (EGS-Ngfp-lacZ (C), EGS-Mtgfp (E), or a mix of EGS-Ngfp-lacZ and EGS-Mtgfp (G)). To locate the nuclei, worms were stained with Hoechst 33258 stain.

To determine the efficacy of the EGSs in inhibiting the expression of their targets, PD4251 worms were treated with EGS-Ngfp-lacZ, EGS-Mtgfp, a mix of EGS-Ngfp-lacZ and EGS-Mtgfp, EGS-Ngfp-lacZ-D, EGS-Mtgfp-D, or a mix of EGS-Ngfp-lacZ-D and EGS-Mtgfp-D. In contrast to worms treated with soaking buffer (Fig. 2A), worms treated with EGS-Ngfp-lacZ (Fig. 2C) or EGS-Mtgfp (Fig. 2E) showed a partial reduction in GFP fluorescence. GFP fluorescence almost disappeared in worms treated with a mix of EGS-Ngfp-lacZ and EGS-Mtgfp (Fig. 2G). By contrast, there was no decrease in GFP fluorescence in worms treated with EGS-Ngfp-lacZ-D (Fig. 2B), EGS-Mtgfp-D (Fig. 2D), or a mix of EGS-Ngfp-lacZ-D and EGS-Mtgfp-D (Fig. 2F). The disabled EGSs were able to serve as negative controls that cannot function effectively for RNase P-based targeting.

**Figure 3:**
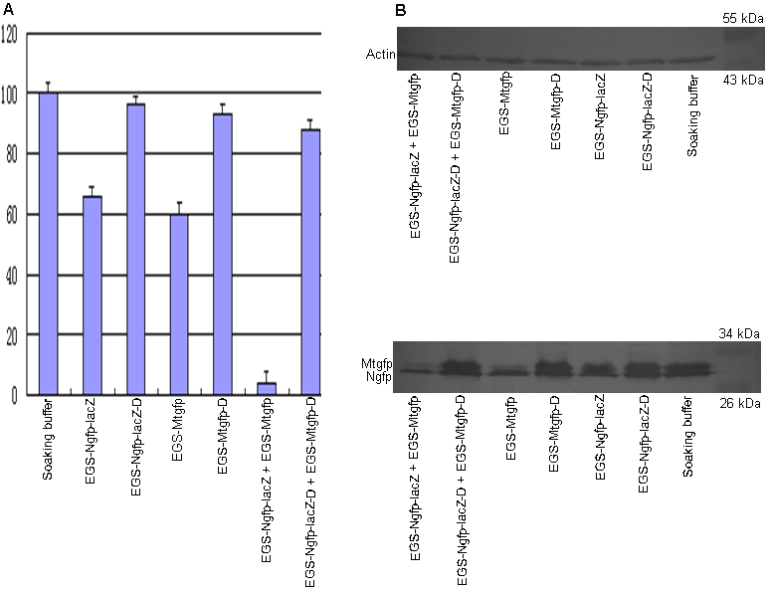
**(A) Effects of EGS on expression levels of GFP mRNA in PD4251 worms. Shown is the GFP mRNA expression level, as measured by QPCR**. (B) Effects of EGS on expression levels of Ngfp and Mtgfp proteins in PD4251 worms treated with EGSs. Shown are the Ngfp and Mtgfp protein expression levels, as measured by Western-blot analysis. Actin protein was used as an internal control.

The expression level of *GFP *mRNA was determined by quantitative PCR (QPCR) analysis (Fig. 3A and Table 1). Reductions of 34% and 40% in the expression level of *GFP *mRNA were observed in worms treated with EGS-Ngfp-lacZ and EGS-Mtgfp, respectively. There was a marked reduction of 96% in the expression level of *GFP *mRNA in worms treated with a mix of EGS-Ngfp-lacZ and EGS-Mtgfp. By contrast, the expression level of *GFP *mRNAs was reduced by <10% in worms treated with EGS-Ngfp-lacZ-D, EGS-Mtgfp-D, or a mix of EGS-Ngfp-lacZ-D and EGS-Mtgfp-D. These results indicate that these EGS-induced significant reductions in the target mRNA expression level were due to RNase P-mediated cleavage. The low level of inhibition in worms treated with these disabled EGSs was presumably due to an anti-sense effect of the EGS.

**Table 1:** Levels of inhibition of the expression of *Ngfp *and *Mtgfp*

Treatment	*GFP *mRNA	Ngfp protein	Mtgfp protein
Soaking buffer	0%	0%	0%
EGS-Ngfp-lacZ	34%	56% ± 5%	5%
EGS-Ngfp-lacZ-D	4%	6%	4%
EGS-Mtgfp	40%	5%	70%
EGS-Mtgfp-D	7%	4%	6%
Mix of EGS-Ngfp-lacZ and EGS-Mtgfp	96%	71% ± 6%	95%
Mix of EGS-Ngfp-lacZ-D and EGS-Mtgfp-D	8%	7%	8%

Inhibition of the expression of *Ngfp *and *Mtgfp *in PD4251 worms that were treated with EGS, compared with the levels of inhibition in PD4251 worms that were treated with soaking buffer. The values shown are means derived from triplicate experiments, and values for the standard deviation that were less than 5% are not shown.

To examine the targeting specificity of EGS-Ngfp-lacZ and EGS-Mtgfp, the protein levels of Ngfp and Mtgfp were determined by Western-blot analysis (Fig. 3B and Table 1). Reductions of 56 ± 5% and less than 10% in the levels of Ngfp and Mtgfp proteins, respectively, were observed in worms treated with EGS-Ngfp-lacZ. Similarly, there were reductions of 70% and less than 10% in the levels of Mtgfp and Ngfp proteins, respectively, in worms treated with EGS-Mtgfp. Interestingly, greater reductions of 71 ± 6% and 95% in the level of Ngfp and Mtgfp proteins, respectively, were observed in worms treated with a mix of EGS-Ngfp-lacZ and EGS-Mtgfp. By contrast, Ngfp and Mtgfp protein levels were reduced by <10% in worms treated with EGS-Ngfp-lacZ-D, EGS-Mtgfp-D or a mix of EGS-Ngfp-lacZ-D and EGS-Mtgfp-D. The small reductions in the Ngfp and Mtgfp protein expression levels in worms treated with these disabled EGSs were likely due to anti-sense effects of the EGSs.

### Generation of EGS library

**Figure 4:**
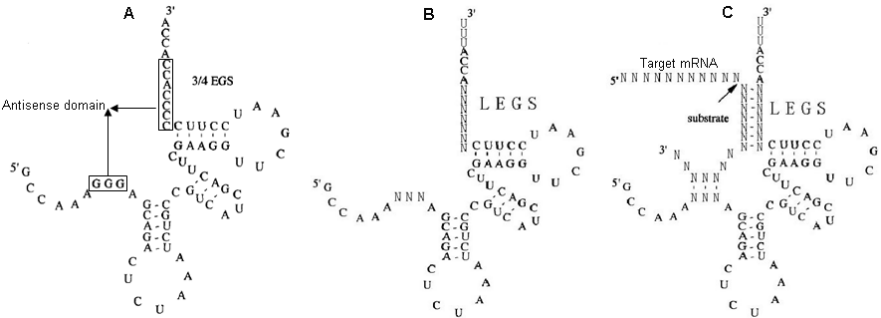
**Demonstration of EGS library**. (A) The "3/4 EGS" is derived from a precursor of tRNA^Tyr ^in *Escherichia coli*. (B) EGS library. The anti-sense domain is composed of random bases. (C) The complex of EGS library and any potential target mRNA. The arrow indicates the RNase P cleavage site.

The "3/4 EGS" (Fig. 4A) was used as a framework for the EGS library. The EGS library (Fig. 4B), which contains a randomized anti-sense domain of the "3/4 EGS", was generated by introducing the following modifications into the framework: the anti-sense domain was composed of random bases; The "CCA" sequence[7,8,28,47,48] located in the 3'-terminus is important for the EGS effect. To protect the "CCA" sequence from being exposed directly to RNase, the "UUU" sequence was attached to its 3'-terminus. The resulting EGS library is a collection that contains any EGS targeted to any target mRNA (Fig. 4C).

**Figure 5:**
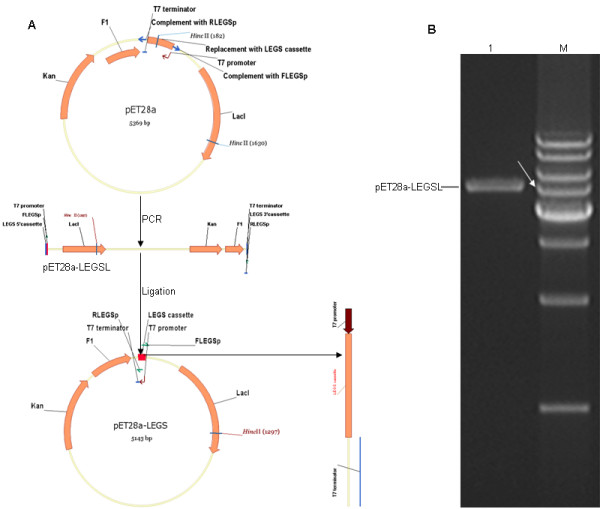
**Construction of pET28a-LEGS**. (A) Flow chart showing the construction of pET28a-LEGS. (B) The PCR product of pET28a-LEGSL (lane 1). The arrow indicates the 5-kb DNA band (lane M).

**Figure 6:**
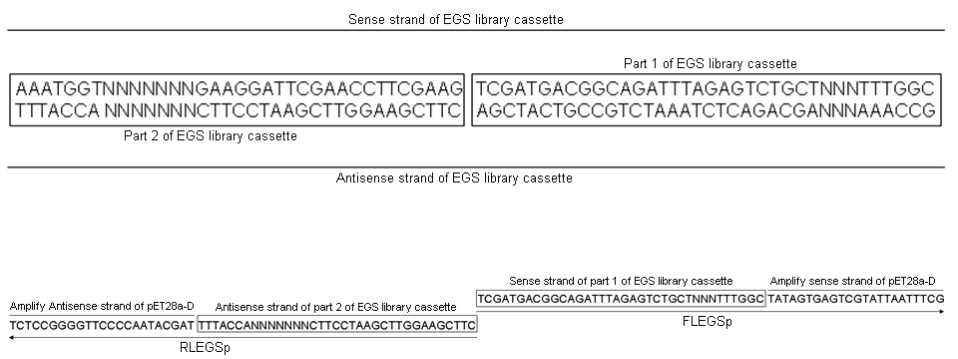
**Demonstration of FLEGSp and RLEGSp.** The partially randomized oligonucleotides of FLESp and RLEGSp are composed of two parts. One is used to amplify pET28a-D, which is equal to pET28a but does not contain the fragment between the T7 terminator and T7 promoter. The other is used to amplify the EGS library cassette.

pET28a-LEGS, which contains the EGS library cassette under control of T7 promoter, was constructed (Fig. 5). First, a primer pair of FLESp and RLEGSp was designed (Fig. 6). The partially randomized oligonucleotides of FLESp and RLEGSp were composed of two parts; one acted as a primer to amplify pET28a-D equal to pET28a but lacked the fragment between the T7 terminator and T7 promoter. The other acted as a primer to amplify the EGS library cassette. Second, pET28a-LEGSL was amplified by PCR with the primer pair of FLEGSp and RLEGSp using pET28a as template. Third, pET28a-LEGS was constructed by self-ligation of pET28a-LEGSL and transformed into DH5α to screen for pET28a-EGS clones containing individual EGS cassettes.

**Figure 7:**
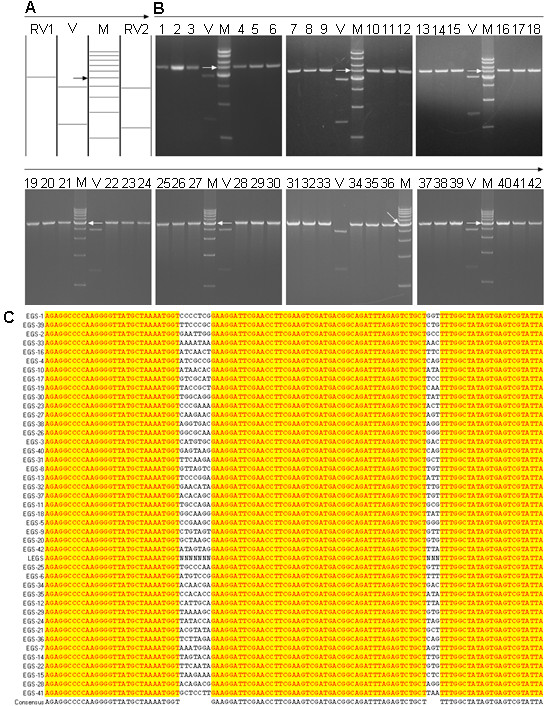
**Examination of composition of the EGS library**. (A) HincII digestion patterns of the pET28a-EGS clone (lane RV1) containing one HincII site, the pET28a-EGS-clone (lane RV2) containing two or three HincII sites, and pET28a (lane V). The arrow indicates the 5-kb DNA band (lane M). (B) The HincII digestion products of the pET28a-EGS clones containing one HincII site (lanes 1–42) and pET28a (lane V). The arrows indicate the 5-kb DNA band (lane M). (C) The alignment of sequences of some EGS cassettes.

In general, about 98% of pET28a-EGS clones have one HincII site, with the remaining 2% having two or three HincII sites. Their HincII digestion patterns were predicted by the NTI program (Fig. 7A). To examine the composition of the EGS library, 500 clones were chosen at random for restriction enzyme (HincII) analysis. Of these 500 clones, 94% (Fig. 7B) showed the HincII digestion pattern shown in Fig. 7A, lane RV1, the rest (see Additional file 1) showed the HincII digestion pattern shown in Fig. 7A, lane RV2. Sequence analysis was performed to determine the specific sequences; 94% were shown to have a unique EGS cassette sequence. Alignment analysis was used to show that these sequences (Fig. 7C) showed no bias in cloning of certain EGS cassettes.

### Validation of EGS library for reverse genetic screen in *C. elegans*

**Figure 8:**
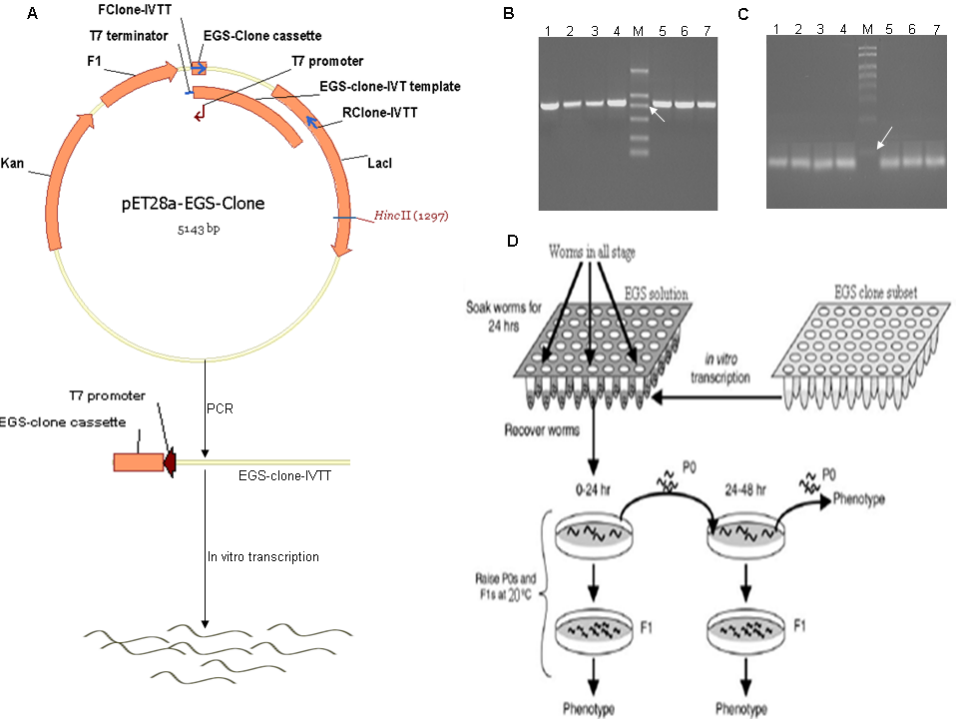
**Reverse genetic screen of *C. elegans *based on EGS**. (A) The flow chart of EGS-clone preparation. (B) The PCR product of the EGS clone IVTT (lanes 1–7). The arrow indicates the 750-bp DNA band (lane M). (C) The transcription product of the EGS clone (lanes 1–7). The arrow indicates the 100-bp RNA band (lane M). (D) Diagram of the phenotype screening procedure.

**Figure 9:**
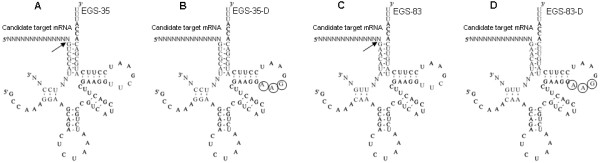
**Complexes between candidate target mRNAs and EGS-35, EGS-35-D, EGS-83, EGS-83-D**. (A, B) Complexes between a candidate target mRNA and EGS-35 (A) and EGS-35-D (B). The arrow indicates the RNase P cleavage site. EGS-35-D was derived from EGS-35 by introducing base-substitution mutations at three positions (highlighted by circles) of the T-loop. (C, D) Complexes between a candidate target mRNA and EGS-83 (C) and EGS-83-D (D). The arrow indicates the RNase P cleavage site. EGS-83-D was derived from EGS-83 by introducing base-substitution mutations at three positions (highlighted by circles) of the T-loop.

To examine whether the EGS library can be used as a reverse genetic screen in *C. elegans*, 300 unique EGSs were randomly selected and used for screening of the *C. elegans *strain N2. The screening procedure is systemically shown in Fig. 8. First, the EGS clone IVTT containing an EGS cassette controlled by the T7 promoter was amplified by PCR with the primers Fclone-IVTT and Rclone-IVTT, using the pET28a-EGS clone as a template (Fig. 8A, B). An EGS clone was transcribed by T7 RNA polymerase using the purified EGS-clone IVTT as a template (Fig. 8A, C). Second, synchronous cultures of N2 worms were soaked in EGS solution. These worms were individually transferred to new plates with food, and phenotypes of both P0 worms and F1 progenies were recorded (Fig. 8D). All phenotypes visible under the dissection microscope were recorded. Such phenotypes included sterility, slow postembryonic growth, larval arrest, larval lethality, abnormal morphology, and uncoordination. About 6% of EGSs induced abnormal phenotypes, such as P0 slow postembryonic growth, P0 larval arrest, P0 larval lethality and P0 sterility (Table 2). Of these, EGS-35 and EGS-83 (Fig. 9A, C) caused the greatest phenotype changes (Table 2). The target mRNAs of EGS-35 and EGS-83 were identified by the following procedure. All candidate target mRNAs of an EGS were identified by a BLAST search of its target sequence (see Additional file 2). BLAST searches of all EGS-35 and EGS-83 candidate target sequences (Table 3) produced 12 and 34 candidate mRNAs (Table 4, 5), respectively. The expression levels of all candidate target mRNAs in worms treated with EGS-35 or EGS-83 were analyzed by QPCR (Tables 6, 7). In worms treated with EGS-35, there were 64% and <10% reductions in the expression levels of ZK858.7 mRNA [Genbank: NM_060051.2] and the other candidate mRNAs, respectively. In worms treated with EGS-83, there were reductions of 72% and <10% in the expression levels of *lin-13 *mRNA [Genbank: NM_066277.3] and the other candidate mRNAs, respectively. By contrast, a reduction of <10% in all candidate target mRNAs was observed in worms treated with EGS-35-D or EGS-83-D (Fig. 9B, D). These small reductions in worms treated with the disabled EGS were likely due to anti-sense effects of the EGSs. These results indicate that the significant reductions in the levels of target mRNA expression (ZK858.7 mRNA and *Lin-13 *mRNA for EGS-35 and EGS-83, respectively) in worms treated with EGSs were due to EGS-directed RNase-P-mediated cleavage. The phenotypes of worms with RNAi-ZK858.7 mRNA and RNAi-*Lin-13 *mRNA were similar to the phenotypes induced by EGS-35 and EGS-83, respectively (Table 2).

**Table 2:** Phenotypes induced by certain EGSs

EGS clone	Phenotype	Target	Corresponding RNAi phenotype
EGS-8	sterile		
EGS-26	sterile		
EGS-29	slow postembryonic growth		
EGS-35	slow postembryonic growth sterile	ZK858.7	Slow growth dumpy sterile progeny embryonic lethal organism morphology abnormal maternal sterile transposon silencing abnormal
			
EGS-41	larval lethality		
EGS-43	sterile		
EGS-80	sterile		
EGS-83	Sterile larval arrest	*Lin-13*	Sterile F1 Larval arrest sterile multivulva organism morphology abnormal protruding vulva
			
EGS-105	larval lethality		
EGS-127	slow postembryonic growth		
EGS-139	slow postembryonic growth		
EGS-156	sterile		
EGS-189	sterile		
EGS-201	larval lethality		
EGS-225	slow postembryonic growth		
EGS-265	larval lethality		
EGS-289	sterile		
EGS-296	sterile		

**Table 3:** Candidate targeting sequences of EGS-35 and EGS-83

EGS	EGS-35	EGS-83
Outline	GTCGCAT**NN**TCC	GAACATA**NN**TTG
All candidate targeting sequences	GTCGCAT**AA**TCC	GAACATA**AA**TTG
	GTCGCAT**AT**TCC	GAACATA**AT**TTG
	GTCGCAT**AG**TCC	GAACATA**AG**TTG
	GTCGCAT**AC**TCC	GAACATA**AC**TTG
	GTCGCAT**TA**TCC	GAACATA**TA**TTG
	GTCGCAT**TT**TCC	GAACATA**TT**TTG
	GTCGCAT**TG**TCC	GAACATA**TG**TTG
	GTCGCAT**TC**TCC	GAACATA**TC**TTG
	GTCGCAT**GA**TCC	GAACATA**GA**TTG
	GTCGCAT**GT**TCC	GAACATA**GT**TTG
	GTCGCAT**GG**TCC	GAACATA**GG**TTG
	GTCGCAT**GC**TCC	GAACATA**GC**TTG
	GTCGCAT**CA**TCC	GAACATA**CA**TTG
	GTCGCAT**CT**TCC	GAACATA**CT**TTG
	GTCGCAT**CG**TCC	GAACATA**CG**TTG
	GTCGCAT**CC**TCC	GAACATA**CC**TTG

The bases marked in bold are not complemented with the EGS

**Table 4:** Candidate target mRNAs of EGS-35

Target mRNA	RNAi phenotype	Targeting sequence
NM_060051.2	slow_growth dumpy sterile_progeny embryonic_lethal organism_morphology_abnormal maternal_sterile transposon_silencing_abnormal	GTCGCATAGTCC
		
NM_076865.3	embryonic_lethal	GTCGCATTTTCC
NM_062956.2	embryonic_lethal	GTCGCATTCTCC
NM_066665.3	embryonic_lethal locomotion_abnormal embryonic_lethal mitotic_spindle_abnormal_early_emb maternal_sterile larval_lethal reduced_brood_size P0_sp	GTCGCATCGTCC
		
NM_001029738.1	fat_content_reduced	GTCGCATCCTCC
NM_072205.2	Norrmal	GTCGCATTTTCC
NM_061028.1	Norrmal	GTCGCATTCTCC
NM_015227.4	No record	GTCGCATTTTCC
NM_001047396.2	No record	GTCGCATGGTCC
NM_001047395.1	No record	GTCGCATGGTCC
NM_074332.2	No record	GTCGCATGGTCC
NM_068962.3	No record	GTCGCATCTTCC

The accession number refers to the GenBank database.

**Table 5:** Candidate target mRNAs of EGS-83

Target mRNA	RNAi phenotype	Targeting sequence
NM_066277.3	sterile_F1 larval_arrest sterile multivulva organism_morphology_abnormal protruding_vulva	GAACATATCTTG
NM_058796.3	male_morphology_abnormal	GAACATATTTTG
NM_066347.2	Abnormal: unclassified phenotypes	GAACATATTTTG
NM_062293.4	Normal	GAACATAAATTG
NM_070343.2	Normal	GAACATAATTTG
NM_074278.2	Normal	GAACATAACTTG
NM_059435.2	Normal	GAACATAACTTG
NM_072012.1	Normal	GAACATATTTTG
NM_059793.2	Normal	GAACATATTTTG
NM_076117.3	Normal	GAACATATATTG
NM_059858.2	Normal	GAACATATATTG
NM_061811.2	Normal	GAACATATATTG
NM_073812.2	Normal	GAACATACCTTG
NM_076627.2	Normal	GAACATACATTG
NM_069246.2	Normal	GAACATACTTTG
NM_061292.2	Normal	GAACATACTTTG
NM_063842.2	Normal	GAACATAGGTTG
NM_072852.1	Normal	GAACATAGTTTG
NM_066363.2	No record	GAACATAAATTG
NM_001028778.1	No record	GAACATAACTTG
NM_171639.2	No record	GAACATATTTTG
NM_171942.1	No record	GAACATATTTTG
NM_171638.2	No record	GAACATATTTTG
NM_001028115.1	No record	GAACATATCTTG
NM_001028116.1	No record	GAACATATCTTG
NM_001028113.1	No record	GAACATATCTTG
NM_001028114.1	No record	GAACATATCTTG
NM_001027086.1	No record	GAACATATCTTG
NM_001027085.1	No record	GAACATATCTTG
NM_001029358.2	No record	GAACATACATTG
NM_001029357.3	No record	GAACATACATTG
NM_001029356.1	No record	GAACATACATTG
NM_001028371.1	No record	GAACATACTTTG
NM_001013620.3	No record	GAACATAGATTG

The accession number refers to the GenBank database.

**Table 6:** QPCR analyses of candidate target mRNAs of EGS-35

Candidate target	Primer for QPCR	Inhibition level
NM_060051.2	5'-AGTCCGGTTTACTCCAAAGCAA-3'	64%
	5'-CCATGAGGCTTTCCAAATGC-3'	
NM_076865.3	5'-TGGCGTTGCAGATAGAATAGGA-3'	8%
	5'-GCCGAAAGCGACATAACCA-3'	
NM_062956.2	5'-CACCAGTAACCCAACAACTCCTAAA-3'	6 ± 5%
	5'-AAGGAGAATGCGACTGGGAAG-3'	
NM_066665.3	5'-TTGAGGCGAAATGCTTGTCA-3'	7%
	5'-TGATGGCAAAATCGATGCA-3'	
NM_001029738.1	5'-TCGGAAACCAGGCAAACAAC-3'	5%
	5'-GGTCATTGTGTGCCATTTCCTT-3'	
NM_072205.2	5'-TTGGTTAGAAGCGAAGTGAGTGA-3'	9%
	5'-AAGGGAGGAGGAAATCAAGAGG-3'	
NM_061028.1	5'-AGAGCACACGGCACATAGGA-3'	5 ± 5%
	5'-CTTGTTCGGGTCTGGGTTG-3'	
NM_015227.4	5'-GCACCTCAGTCTCAACATTTTCTTT-3'	7%
	5'-TCACACGCCTTCTCTTGGTCT-3'	
NM_001047396.2	5'-GCTCCGATTCAAGTCATGTGG-3'	7 ± 5%
	5'-GCAAGCCGAAGAGGTGATGT-3'	
NM_001047395.1	5'-AGGAACACCAATGGTCACAATG-3'	7%
	5'-GGAACTCCGAGAGCGTAAAGCT-3'	
NM_074332.2	5'-TCGTTCTGTCACGGGGAAC-3'	5 ± 6%
	5'-CTTCGCATCTTTTCCACCAAC-3'	
NM_068962.3	5'-CATCGTCATCTAGTCTCCCAGTGT-3'	8%
	5'-TTACTTCGTTTGGTTGGTGGTG-3'	

The accession number refers to the GenBank database. The values shown are means derived from triplicate experiments, and values for the standard deviation that were less than 5% are not shown.

**Table 7:** Primer pairs for QPCR analysis candidate target mRNAs of EGS-83

Candidate target	Primer for QPCR	Inhibition level
NM_066277.3	5'-AATCGAAAGCTCCGTTATCCAC-3'	72%
	5'-TTCCCTCGGCTTCCAAAA-3'	
NM_058796.3	5'-TAAACGTGGCGGAGCTATCG-3'	5%
	5'-CGTTCTCAATGCCCTTCCA-3'	
NM_066347.2	5'-AAAATCATTGGTCCCGTCATG-3'	6%
	5'-CCAACCAAGAAGGGCATTCA-3'	
NM_062293.4	5'-AAGAGATGGATGTCTGGTAGTGGA-3'	5 ± 6%
	5'-GGAAGAGAGCATCGTTTTGGA-3'	
NM_070343.2	5'-AAGGACGGGAGGAACTGGA-3'	6 ± 5%
	5'-TTGGGAACGAGGGAACACTT-3'	
NM_074278.2	5'-ACCTTCTTGTGCCAATATTTGGA-3'	8%
	5'-TTGCCATGAAGTTTCCGAAAA-3'	
NM_059435.2	5'-TGGCGTCCGTTACCTTGAA-3'	6%
	5'-GAATCAGCGGAGAATGCACAT-3'	
NM_072012.1	5'-CCATTGGACATGGGAAAAACA-3'	5%
	5'-TGGGATATTGGATTTTTGGTCAA-3'	
NM_059793.2	5'-GGCACTTTTGTTGCGATTGAA-3'	6 ± 5%
	5'-GGCTCTACAAGTTCCCAGCAAAT-3'	
NM_076117.3	5'-TGCAATTATGGTGCACTGATAACA-3'	8%
	5'-TTGCGACATTTTCGAATCGA-3'	
NM_059858.2	5'-GCGATGGTATTTTTGGCAGAA-3'	6%
	5'-TTCTCCGTATCCGCACTTGAA-3'	
NM_061811.2	5'-TTTCAGAGTTTACCCGATGTTCAG-3'	5%
	5'-CCGTATTTCCCGTAGTTTGAGG-3'	
NM_073812.2	5'-CCGAAGCGTCTGTATTAGTTGCT-3'	5%
	5'-TTTGACTTTTGCGGTGGATG-3'	
NM_076627.2	5'-GGGAGCAGTTGTGAGAGGATTT-3'	7 ± 5%
	5'-CCGCCTTCTCCGTCTTCTT-3'	
NM_069246.2	5'-GCTCTGGTCGCTACTCAATCAA-3'	9%
	5'-ATTACTTCCTTGTGCCTCCATCTC-3'	
NM_061292.2	5'-CGCAAAACTCGGGCAAA-3'	6%
	5'-GCCGTAGCCTCCATCAAAAC-3'	
NM_063842.2	5'-TCGTCACATTTTCCGTTTCTCA-3'	7%
	5'-GACCTGCTCCCCTGACAGTAGT-3'	
NM_072852.1	5'-GGCTGAAACCAAGAACGAAAA-3'	7%
	5'-TCGCAGAAGGAATGGAAGTG-3'	
NM_066363.2	5'-TGAAAGCTGGCGAAGGACTT-3'	5 ± 5%
	5'-CAAGGGTTTCCAACGCAAAT-3'	
NM_001028778.1	5'-CGAAGCGAACGGGATAATAGTG-3'	6%
	5'-CGACTCATGTGCAAGTTATGTTCTT-3'	
NM_171639.2	5'-CGAGGATGTTGCCATTCAGTT-3'	7%
	5'-GAAGATTTGGGTTTTCACCATGA-3'	
NM_171942.1	5'-TCAGATCACTCATGAACTCCATGA-3'	5%
	5'-GAAGGGCGAAAATGAGAATGAA-3'	
NM_171638.2	5'-CGGTGGAAGAGATGGATGAAG-3'	6%
	5'-TTCGTGATTCGGTGGAACAA-3'	
NM_001028115.1	5'-GCACAACTTTATCGCAACGATTA-3'	6%
	5'-GCAAAGCTGGTGCAATTCTTC-3'	
NM_001028116.1	5'-CTGCCTTTGCCGATGGTTA-3'	6%
	5'-CTGGATAGTAGTAGGGCTCCGAAA-3'	
NM_001028113.1	5'-CTGCCTTTGCCGATGGTTA-3'	6%
	5'-CTGGATAGTAGTAGGGCTCCGAAA-3'	
NM_001028114.1	5'-CTGCCTTTGCCGATGGTTA-3'	6%
	5'-CTGGATAGTAGTAGGGCTCCGAAA-3'	
NM_001027086.1	5'-GGAGCGCGTCAGAGTAAACG-3'	7%
	5'-TTTCGAGACGGCCTTTGTTC-3'	
NM_001027085.1	5'-GGAGCGCGTCAGAGTAAACG-3'	7%
	5'-TTTCGAGACGGCCTTTGTTC-3'	
NM_001029358.2	5'-ATGCATATTGAGCACGCAGAA-3'	5%
	5'-CGGGCAGATGCAATTGTTT-3'	
NM_001029357.3	5'-CGAGAGCGGCGAGTTGATAG-3'	5%
	5'-ATACTGCATCCGAGCAACATGT-3'	
NM_001029356.1	5'-CGAGAGCGGCGAGTTGATAG-3'	5%
	5'-ATACTGCATCCGAGCAACATGT-3'	
NM_001028371.1	5'-CCCAAATGTTATGCCAGTCAAG-3'	5%
	5'-TTGGAAGATGTAGAATGGTGAGAGA-3'	
NM_001013620.3	5'-CAGACTTCCACCTATTAAAGGACCA-3'	7%
	5'-CACAAAACAGAAATCCCAGAAGG-3'	

The accession number refers to the GenBank database. The values shown are means derived from triplicate experiments, and values for the standard deviation that were less than 5% are not shown.

## Discussion

It has been shown that EGS technology can be used to down-regulate gene expression in bacteria [9-12], mammalian cells [13-19] and maize cells[20]. We have shown that EGS technology can also be used to down-regulate gene expression in *C. elegans*. Several criteria must be satisfied if successful EGS targeting is to be achieved. Among these are high cleavage efficiency, EGS target specificity, and efficient delivery of the reagent. We constructed EGS-Ngfp-lacZ and EGS-Mtgfp that target *Ngfp-lacZ *and *Mtgfp *mRNAs, respectively, and showed that these EGSs direct RNase P to cleave the targets efficiently. Moreover, we showed targeting specificity of these EGSs. Although the complementary sequence of anti-sense domain of EGS-Ngfp-lacZ is present in *Mtgfp *mRNA, a small reduction in the Mtgfp protein expression level was observed in worms treated with EGS-Ngfp-lacZ. In addition, *Ngfp-lacZ *mRNA contains the complementary sequence of the anti-sense domain of EGS-Mtgfp, but there was a small reduction in the Ngfp protein expression level in worms treated with EGS-Mtgfp. Reduction of <10% in the expression levels of Ngfp and Mtgfp proteins were observed in worms treated with EGS-Ngfp-lacZ-D or EGS-Mtgfp-D, respectively. Together, these results indicate that the significant reductions in the Ngfp and Mtgfp protein expression levels in worms that treated with EGS-Ngfp-lacZ and EGS-Mtgfp, respectively, were primarily due to EGS-induced RNase-P-targeted cleavage rather than anti-sense effects or other nonspecific effects of the EGSs. The mix of EGS-Ngfp-lacZ and EGS-Mtgfp had greater effects on inhibition of *Ngfp-lacZ *and *Mtgfp *expression compared with individual EGS-Ngfp-lacZ and individual EGS-Mtgfp, respectively (Fig. 3B and Table 1). This was probably due to anti-sense effects of the EGSs, but is not due to any overlap in the target sequence. Maybe the EGS methodology is particularly effective when more than one site in a particular mRNA is targeted[12,16].

Many *C. elegans *genes have been associated with phenotypes due to the results of reverse genetic screens based on RNAi libraries. Despite the success of these screens, the functions of most of approximately 20,000 predicted genes in the *C. elegans *genome remain elusive. Moreover, the limitations of RNAi such as off-target [52-54] and relative variability in the RNAi effect[40] compromise the level of confidence in the results of these RNAi screens. The EGS library aims to facilitate reverse genetic screens such as those with the RNAi library, and it will be useful for confirming RNAi phenotypes. For example, ZK858.7 and *lin-13 *genes were identified by a reverse genetic screen based on the EGS library. Remarkably, EGS-35 and EGS-83 efficiently and specifically interfered with ZK858.7 and *lin-13*, respectively. The target specificity of the EGS is governed by two different types of interactions[3,19]. One is the base-pairing interactions[3,17,19,55] in which the ten nucleotides in the EGS hybridize with the accessible region of the target mRNA. The EGS has two short, sequence-specific recognition elements that are oriented in space with respect to each other in a well-defined fashion. This complex recognition element provides the necessary specificity for RNase P. It is known that the ten nucleotides involved in base-pairing between the EGS and the target mRNA make it difficult to guarantee target specificity in *C. elegans*. Given the extensive secondary and tertiary structure associated with the RNA or the binding of proteins to the target RNA *in vivo*, the target sequences in cellular RNAs are not all accessible. The other type of interaction[3,17,19,55] is between the RNase P recognition domain (e.g., T-loop and stem) and the mRNA. This interaction facilitates the folding of the EGS-mRNA complex into a tRNA-like molecule and stabilizes the mRNA-EGS complex. An immediate corollary is that if two targets with a one-bp mismatch are compared, the same caveat on accessibility rules out any meaningful comment on specificity of targeting. Mutation of a single base in the target mRNA will not affect the methodology based on "stem EGS" because a single base mismatch in the complex with the target mRNA is unlikely to alter recognition by RNase P[9,12]. However, the location of the unpaired nucleotides is important because three contiguous unpaired bases might very well disallow the RNase P-mediated effects. It is that an EGS could still function despite several point mutations between it and the bacterial target mRNA, depending precisely on the sequence of the unpaired bases[9]. The framework of EGS-35 and EGS-83 is the "3/4 EGS" that is distinguishable from the "stem EGS" by additional parts equivalent to the T-stem and T-loop, and variable regions of a tRNA. The mismatch tolerance of the effects of EGS-35 and EGS-83 needs further study. Since the worms are cultured at 20°C, specificity considerations for antisense-based techniques are different compared to plants/animals whose growth temperatures range from 25 to 37°C.

## Conclusion

EGS technology can be used to interfere with gene expression in *C. elegans*. The EGS library is used to facilitate a reverse genetic screen as performed by a RNAi library, and it should be particularly useful for confirming the RNAi phenotype as the function of most of the approximately 20,000 predicted genes in the *C. elegans *genome remains elusive. Moreover, the limitations of RNAi such as off-target and relative variability in the RNAi effect compromise the level of confidence in the RNAi screen results. Taken together, these observations are potentially of great importance for further our understanding and promoting the development of *C. elegans *genomics.

## Methods

### *C. elegans*, primers and vector

The N2 and PD4251 strains of *C. elegans *were provided by the *Caenorhabditis *Genetics Center (Univ. of Minnesota, St. Paul). The worms were maintained and handled as described previously[56]. Primers used in this work are listed in Table 8. The pET28a vector was purchased from Merk, Inc.

**Table 8:** Primers used in this study

Primer	Sequence
FLEGSp	5'-pTCGATGACGGCAGATTTAGAGTCTGCTNNNTTTGGCTATAGTGAGTCGTATTAATTTCG-3'
RLEGSp	5'-pCTTCGAAGGTTCGAATCCTTCNNNNNNNACCATTTTAGCATAACCCCTTGGGGCCTCT-3'
FEGSp-Ngfp-lacZ	5'p-TCGATGACGGCAGATTTAGAGTCTGCTTTCTTTGGCTATAGTGAGTCGTATTAATTTCG-3'
REGSp-Ngfp-lacZ	5'p-CTTCGAAGGTTCGAATCCTTCTTTAAATACCATTTTAGCATAACCCCTTGGGGCCTCT-3'
FEGSp-Mtgfp	5'p-TCGATGACGGCAGATTTAGAGTCTGCTATCTTTGGCTATAGTGAGTCGTATTAATTTCG-3'
REGSp-Mtgfp	5'p-CTTCGAAGGTTCGAATCCTTCTATTAACACCATTTTAGCATAACCCCTTGGGGCCTCT-3'
FEGSp-Ngfp-lacZ-D	5'p-TCGATGACGGCAGATTTAGAGTCTGCTTTCTTTGGCTATAGTGAGTCGTATTAATTTCG-3'
REGSp-Ngfp-lacZ-D	5'p-CTTCGAAGG**AAG**GAATCCTTCTTTAAATACCATTTTAGCATAACCCCTTGGGGCCTCT-3'
FEGSp-Mtgfp-D	5'p-TCGATGACGGCAGATTTAGAGTCTGCTATCTTTGGCTATAGTGAGTCGTATTAATTTCG-3'
REGSp-Mtgfp-D	5'p-CTTCGAAGG**AAG**GAATCCTTCTATTAACACCATTTTAGCATAACCCCTTGGGGCCTCT-3'
FLEGSp-35-D	5'-pTCGATGACGGCAGATTTAGAGTCTGCTTCCTTTGGCTATAGTGAGTCGTATTAATTTCG-3'
RLEGSp-35-D	5'-pCTTCGAAGG**AAG**GAATCCTTCATGCGACACCATTTTAGCATAACCCCTTGGGGCCTCT-3'
FLEGSp-83-D	5'-pTCGATGACGGCAGATTTAGAGTCTGCTTTGTTTGGCTATAGTGAGTCGTATTAATTTCG-3'
RLEGSp-83-D	5'-pCTTCGAAGG**AAG**GAATCCTTCTATGTTCACCATTTTAGCATAACCCCTTGGGGCCTCT-3'
Fclone-IVTT	5'-AAATGGTNNNNNNNGAAG-3'
Rclone -IVTT	5'-AGATTGTGCACCGCCGCT-3'
FNgfp-lacZ-IVTT	5'-AAATGGTATTTAAAGAAGGA-3'
RNgfp-lacZ-IVTT	5'-AGATTGTGCACCGCCGCT-3'
FMtgfp-IVTT	5'-AAATGGTGTTAATAGAAGGA-3'
RMtgfp-IVTT	5'-AGATTGTGCACCGCCGCT-3'
F35-IVTT	5'-AAATGGTGTCGCATGAAG-3'
R35 -IVTT	5'-AGATTGTGCACCGCCGCT-3'
F83-IVTT	5'-AAATGGTGAACATAGAAG-3'
R83 -IVTT	5'-AGATTGTGCACCGCCGCT-3'
eft-2-QPCR-F	5'-GACGAGAAGGATTTGGAAGGAA-3'
eft-2-QPCR-R	5'-ACTGGGGATGGAAGATGGAA-3'
GFP-QPCR-F	5'-TGGAGTTGTCCCAATTCTTGTT-3'
GFP-QPCR-R	5'-GCATCACCTTCACCCTCTCC-3'
S-EGS-F	5'-TTAGAGCTTGACGGGGAAAG-3'
S-EGS-R	5'-CCTGCCACCATACCCACGCC-3'

The "p" in the "sequence" column represents the modification by phosphorylation. Fclone-IVTT is the outline of the corresponding primer used in the specific experiment. Base-substitution mutations at three positions of the T-loop are indicated by bold text.

### Synchronous cultures of *C. elegans*

Synchronous cultures of *C. elegans *were prepared basically as described previously[56]. The worms were washed well in M9 solution (43.6 mM Na_2_HPO_4_, 22.0 mM KH_2_PO_4_, 8.6 mM NaCl, and 18.7 mM NH_4_Cl)[34] to completely remove bacteria. Then, they were starved and washed well in 0.25 × M9 solution.

### Preparations of EGS-Ngfp-lacZ, EGS-Mtgfp

The EGSs that specifically target *Ngfp-lacZ *or *Mtgfp *mRNAs are designed using RNA-folding software[46]. According to the rules of EGS design[28], the favorable accessible regions of *Ngfp-lacZ *(Fig. 1A) and *Mtgfp *mRNAs (Fig. 1B) were identified from all candidate accessible regions. The "3/4 EGS" (Fig. 1C) was used as the design framework. The anti-sense sequence of the accessible region was introduced into the anti-sense domain of the design framework. The "CCA" sequence[7,8,28,47,48] located in the 3'-terminus is important for the EGS effect. To protect the "CCA" sequence from being exposed directly to RNase, the "UUU" sequence was attached to its 3'-terminus. To construct pET28a-EGS-Ngfp-lacZ and pET28a-Mtgfp, which contain EGS-Ngfp-lacZ and EGS-Mtgfp cassettes, respectively, under the control of the T7 promoter, primer pairs were designed using the NTI program (see Additional file 3) and synthesized with 5'-terminal phosphorylation modification. The pET28a-EGS-Ngfp-lacZ and pET28a-Mtgfp constructs were generated (see Additional files 4, 5). pET28a-EGS-Ngfp-lacZL was amplified by PCR with the primer pair of FEGSp-Ngfp-lacZ and REGSp-Ngfp-lacZ using pET28a as a template; the reaction conditions were 98°C for 60 s, 30 cycles of 98°C for 5 s, 70°C for 15 s and 72°C for 90 s, followed by 72°C for 10 min, in 50-μl volumes with Phusion DNA Polymerase (NEB: F-530S). PCR amplification of pET28a-EGS-MtgfpL was performed as described above using the primer pair of FEGSp-Mtgfp and REGSp-Mtgfp. The purified PCR products were self-ligated by T4 ligase (NEB) at 15°C for 16 hours. The ligation product was transformed into DH5α-competent cells and screened with 30 μg/ml kanamycin. The recombinant vectors of pET28a-EGS-Ngfp-lacZ and pET28a-EGS-Mtgfp were identified by restriction enzyme digest with HincII and sequencing with the S-LEGS-F or S-LEGS-R primers. *In vitro *transcriptions of EGS-Ngfp-lacZ and Mtgfp were demonstrated (see Additional file 6). PCR amplification of EGS-Ngfp-lacZ-IVTT was performed with the primer pair of FNgfp-lacZ-IVTT and RNgfp-lacZ-IVTT using pET28a-EGS-Ngfp-lacZ as a template; the reaction conditions were 98°C for 60 s, 30 cycles of 98°C for 5 s, 70°C for 15 s, and 72°C for 15 s, followed by 72°C for 10 min, in 50-μl volumes with Phusion DNA Polymerase (NEB: F-530S). PCR amplification of EGS-Mtgfp-IVTT was performed as described above but using pET28a-EGS-Mtgfp as a template and the primer pair of RMtgfp-IVTT and FMtgfp-IVTT. EGS-Ngfp-lacZ and EGS-Mtgfp were transcribed *in vitro *by T7 RNA polymerase (Epicentre) using purified PCR products of EGS-Ngfp-IVTT and EGS-Mtgfp-IVTT, respectively, as templates.

### Preparations of EGS-Ngfp-lacZ-D, EGS-Mtgfp-D, EGS-35-D and EGS-83-D

EGS-Ngfp-lacZ-D, EGS-Mtgfp-D, EGS-35-D and EGS-83-D were derived from EGS-Ngfp-lacZ, EGS-Mtgfp, EGS-35 and EGS-83, respectively, and contained point mutations (5'-TTC-3' → AAG) at the three highly conserved positions in the "T-loop" of these EGSs. These nucleotides are found in most of the known natural tRNA sequences[49] and are thought to be important for the interactions between the tRNA domains and human RNase P[3]. Previous studies have shown that EGSs with these mutations prevented RNase P recognition and exhibited little activity in directing RNase-P-mediated cleavage[19,50,51]. EGS-Ngfp-lacZ-D, EGS-Mtgfp-D, EGS-35-D and EGS-83-D were constructed according to the same strategy as described above. For full details, see additional files 7, 8, 9, 10, 11, 12, 13, 14, 15.

### Validation of effectiveness of EGS-Ngfp-lacZ and EGS-Mtgfp

The purified EGSs were dissolved in 400 μl soaking buffer (10.9 mM Na_2_HPO_4_, 5.5 mM KH_2_PO_4_, 2.1 mM NaCl, 4.7 mM NH_4_Cl, 6 mM spermidine, and 0.1% gelatin)[34]. The final RNA concentration varied from 6 to 10 mg/ml. Synchronous cultures of *C. elegans *strain PD4251 (containing 400 L1 larvae, 400 L2 larvae, 400 L3 larvae and 400 L4 larvae) in volumes of 400 μl 0.25 × M9 solution were added to EGS solution and shaken at 20°C for 24 hours. The treated worms underwent the following analyses: GFP fluorescence of PD4251 worms was imaged by microscope; to locate the nuclei, and worms were stained with Hoechst 33258 (sigma) according to standard protocol. Total RNA was prepared as described in the "Experimental Procedures and Protocols for Total RNA Isolation" developed and provided by Stuart Kim's laboratory. Primers for quantitative real-time PCR (QPCR) were: *eft-2 *(eft-2-QPCR-F and eft-2-QPCR-R) and *GFP *(GFP-QPCR-F and GFP-QPCR-R). QPCR was performed using PrimeScript™ RT reagent kit and PrimeScript^® ^*Premix Ex Taq*™ kit (TAKARA) according to the manufacturer's instructions. Expression level of *GFP *mRNA was normalized to the mRNA *eft-2 *expression level. Protein was prepared according to the "Protocol of Protein prep from *C. elegans *and Western Analysis" provided by the Pasquinelli laboratory. Western-blot analysis was performed using the following antibodies: actin (I-19)(SANTA CRUZ sc-1616), GFP (B-2) (SANTA CRUZ sc-9996), bovine anti-mouse IgG-AP (SANTA CRUZ sc-2373), and donkey anti-goat IgG-AP (SANTA CRUZ sc-2022). The films were imaged using the UVP gel imaging analytical system (Upland, GDS-8000) and analyzed using Labworks software. Actin protein was used as an internal control.

### Construction of EGS library

To construct pET28a-LEGS, which contains the EGS library cassette under control of the T7 promoter, the primer pair of FLEGSp and RLEGSp was designed using the NTI program and synthesized with random bases at certain positions and 5'-terminal phosphorylation modifications. pET28a-LEGSL was amplified by PCR with the primer pair of FEGSp and REGSp using pET28a as a template; the reaction conditions were 98°C for 60 s, 30 cycles of 98°C for 5 s, 70°C for 15 s and 72°C for 90 s, followed by 72°C for 10 min, in 50-μl volumes with Phusion DNA Polymerase (NEB: F-530S). One microgram of the purified PCR product of pET28a-LEGSL was self-ligated by T4 ligase (NEB) in a 1-ml volume at 15°C for 16 hours. The ligation product was purified and transformed into DH5α maximum efficiency competent cells (Invitrogen: 18258-012), and selection of bacterial clones was performed with 30 μg/ml kanamycin. Individual clones were selected at random for restriction enzyme digest with HincII and sequencing with the S-LEGS-F or S-LEGS-R primers.

### Reverse genetic screen based on EGS

PCR amplification of the EGS clone IVTT was performed with the primer pair of Fclone-IVTT and Rclone-IVTT using the pET28a-EGS clone as a template; the reaction conditions were: 98°C for 60 s, 30 cycles of 98°C for 5 s, 70°C for 15 s and 72°C for 15 s, followed by 72°C for 10 min, in 50-μl volume with Phusion DNA Polymerase (NEB: F-530S). The EGS clone was transcribed *in vitro *using T7 RNA polymerase (Epicentre) and the purified PCR product of the EGS clone IVTT as a template. The purified EGS clone was dissolved in 4 μl soaking buffer (10.9 mM Na_2_HPO_4_, 5.5 mM KH_2_PO_4_, 2.1 mM NaCl, 4.7 mM NH_4_Cl, 6 mM spermidine, and 0.1% gelatin)[34]. The final RNA concentration varied from 6 to 10 mg/ml. Purified synchronous cultures of *C. elegans *strain N2 (containing 3 L1 larvae, 3 L2 larvae, 3 L3 larvae and 3 L4 larvae) in a volume of 4 μl 0.25 × M9 solution were added to each EGS solution in 48-well PCR plates and shaken at 20°C for 24 hours. The worms were then transferred to new plates with food and phenotypes of both P0 worms and F1 progenies were recorded.

### Identification of target mRNA of EGS-35 and EGS-83

All candidate target mRNAs of an EGS were identified by BLAST analysis of the target sequence (see Additional file 2). The expression levels of all candidate target mRNAs in worms treated with EGS-35 or EGS-83 were analyzed by QPCR as described above. Primers for QPCR are listed in Tables 6, 7 and 8. Expression levels of candidate target mRNA were normalized to the expression level of the mRNA *eft-2*.

## Authors' contributions

QY validated EGS technology in *C. elegans*, generated the EGS library, performed the reverse genetic screen and wrote the first draft of the manuscript. RZ generated the EGS library, performed the reverse genetic screen and participated in writing the manuscript. CY performed the reverse genetic screen. BZ performed the Western-blot analysis. WZ and WM initiated the project, designed the EGS library and finalized writing the manuscript. All authors read and approved the final manuscript.

## Supplementary Material

Additional File 1 **HincII digestion pattern**. The HincII digestion products of the pET28a-EGS clone (lane 1) containing two or three HincII sites, the pET28a-EGS clones (lanes 2–6) containing one HincII site, and pET28a (lane V). The arrow indicates the 5-kb DNA band (lane M). Click here for file

Additional File 2 **All candidate target mRNAs of an EGS were identified by BLAST analysis of the target sequence**. The NCBI-BLAST web interface. Click here for file

Additional File 3 **Demonstration of primer pair**. (A) The primer pair of FEGSp-Ngfp-lacZ and REGSp-Ngfp-lacZ. The partially randomized oligonucleotides of FEGSp-Ngfp-lacZ and REGSp-Ngfp-lacZ were composed of two parts. One was used to amplify pET28a-D, which is equal to pET28a but without the fragment between the T7 terminator and T7 promoter. The other was used to amplify the EGS-Ngfp-lacZ cassette. (B) The primer pair of FEGSp-Mtgfp and REGSp-Mtgfp. The partially randomized oligonucleotides of FEGSp-Mtgfp and REGSp-Mtgfp were composed of two parts. One was used to amplify pET28a-D; the other was used to amplify EGS-Mtgfp cassette. Click here for file

Additional File 4 **Construction of pET28a-EGS-Ngfp-lacZ**. (A) Flow chart showing construction of pET28a-EGS-Ngfp-lacZ. (B) The PCR product of pET28a-EGS-Ngfp-lacZL (lane 1). The arrow indicates the 5-kb DNA band (lane M). Click here for file

Additional File 5 **Construction of pET28a-EGS-Mtgfp**. (A) Flow chart showing pET28a-EGS-Mtgfp construction. (B) The PCR product of pET28a-EGS-MtgfpL (lane 1). The arrow indicates the 5-kb DNA band (lane M). Click here for file

Additional File 6 **Preparations of EGS-Ngfp-lacZ and EGS-Mtgfp**. (A) Flow chart showing preparation of EGS-Ngfp-lacZ. (B) Flow chart showing preparation of EGS-Mtgfp. (C) The PCR products of IVTT-EGS-Ngfp-lacZ (lane 1) and IVTT-EGS-Mtgfp (lane 2). The arrow indicates the 750-bp DNA band (lane M). (D) The transcription products of EGS-Ngfp-lacZ (lane 1) and EGS-Mtgfp (lane2). The arrow indicates the 100-bp RNA band (lane M). Click here for file

Additional File 7 **Demonstration of primer pair**. (A) The primer pair of FEGSp-Ngfp-lacZ-D and REGSp-Ngfp-lacZ-D. The partially randomized oligonucleotides of FEGSp-Ngfp-lacZ-D and REGSp-Ngfp-lacZ-D were composed of two parts. One was used to amplify pET28a-D, which is equal to pET28a but without the fragment between the T7 terminator and T7 promoter. The other was used to amplify the EGS-Ngfp-lacZ-D cassette. (B) The primer pair of FEGSp-Mtgfp-D and REGSp-Mtgfp-D. The partially randomized oligonucleotides of FEGSp-Mtgfp-D and REGSp-Mtgfp-D were composed of two parts. One was used to amplify pET28a-D; the other was used to amplify EGS-Mtgfp-D cassette. Click here for file

Additional File 8 **Demonstration of primer pair**. (A) The primer pair of FEGSp-35-D and REGSp-35-D. The partially randomized oligonucleotides of FEGSp-35-D and REGSp-35-D were composed of two parts. One was used to amplify pET28a-D, which is equal to pET28a but without the fragment between the T7 terminator and T7 promoter. The other was used to amplify the EGS-35-D cassette. (B) The primer pair of FEGSp-83-D and REGSp-83-D. The partially randomized oligonucleotides of FEGSp-83-D and REGSp-83-D were composed of two parts. One was used to amplify pET28a-D; the other was used to amplify the EGS-83-D cassette. Click here for file

Additional File 9 **Construction of pET28a-EGS-Ngfp-lacZ-D**. (A) Flow chart showing construction of pET28a-EGS-Ngfp-lacZ-D. (B) The PCR product of pET28a-EGS-Ngfp-lacZ-DL (lane 1). The arrow indicates the 5-kb DNA band (lane M). Click here for file

Additional File 10 **Construction of pET28a-EGS-Mtgfp-D**. (A) Flow chart showing construction pET28a-EGS-Mtgfp-D. (B) The PCR product of pET28a-EGS-Mtgfp-DL (lane 1). The arrow indicates the 5-kb DNA band (lane M). Click here for file

Additional File 11 **Preparations of EGS-Ngfp-lacZ-D and EGS-Mtgfp-D**. (A) Flow chart showing preparation of EGS-Ngfp-lacZ-D. (B) Flow chart showing preparation of EGS-Mtgfp-D. (C) The PCR products of IVTT-EGS-Ngfp-lacZ-D (lane 1) and IVTT-EGS-Mtgfp-D (lane 2). The arrow indicates the 750-bp DNA band (lane M). (D) The transcription products of EGS-Ngfp-lacZ-D (lane 1) and EGS-Mtgfp-D (lane2). The arrow indicates the 100-bp RNA band (lane M). Click here for file

Additional File 12 **Preparations of EGS-35 and EGS-83**. (A) Flow chart showing preparation of EGS-35. (B) Flow chart showing preparation of EGS-83. (C) The PCR products of IVTT-EGS-35 (lane 1) and IVTT-83 (lane 2). The arrow indicates the 750-bp DNA band (lane M). (D) The transcription products of EGS-35 (lane 1) and EGS-83 (lane 2). The arrow indicates the 100-bp RNA band (lane M). Click here for file

Additional File 13 **Construction of pET28a-EGS-35-D**. (A) Flow chart showing construction of pET28a-EGS-35-D. (B) The PCR product of pET28a-EGS-35-DL (lane 1). The arrow indicates the 5-kb DNA band (lane M). Click here for file

Additional File 14 **Construction of pET28a-EGS-83-D**. (A) Flow chart showing construction of pET28a-EGS-83-D. (B) The PCR product of pET28a-EGS-83-DL (lane 1). The arrow indicates the 5-kb DNA band (lane M). Click here for file

Additional File 15 **Preparations of EGS-35-D and EGS-83-D**. (A) Flow chart showing preparation of EGS-35Z-D. (B) Flow chart showing preparation of EGS-83-D. (C) The PCR products of IVTT-EGS-35-D (lane 1) and IVTT-EGS-83-D (lane 2). The arrow indicates the 750-bp DNA band (lane M). (D) The transcription products of EGS-35-D (lane 1) and EGS-83-D (lane 2). The arrow indicates the 100-bp RNA band (lane M). Click here for file

## References

[B1] Frank DN, Pace NR (1998). Ribonuclease P: unity and diversity in a tRNA processing ribozyme. Annu Rev Biochem.

[B2] Trang P, Kim K, Liu F (2004). Developing RNase P ribozymes for gene-targeting and antiviral therapy. Cell Microbiol.

[B3] Altman S, Kirsebom LA, Raymond F Gesteland, Thomas R Cech, John F Atkins (2006). Ribonuclease P: Structure and Catalysis. The RNA World University of Utah, Salt Lake City; University of Colorado, Boulder; University of Utah, Salt Lake City).

[B4] Gopalan V, Vioque A, Altman S (2002). RNase P: variations and uses. J Biol Chem.

[B5] Walker SC, Engelke DR (2006). Ribonuclease P: the evolution of an ancient RNA enzyme. Crit Rev Biochem Mol Biol.

[B6] Li Y, Guerrier-Takada C, Altman S (1992). Targeted cleavage of mRNA in vitro by RNase P from Escherichia coli. Proc Natl Acad Sci USA.

[B7] Forster AC, Altman S (1990). External guide sequences for an RNA enzyme. Science.

[B8] Yuan Y, Hwang ES, Altman S (1992). Targeted cleavage of mRNA by human RNase P. Proc Natl Acad Sci USA.

[B9] McKinney J, Guerrier-Takada C, Wesolowski D, Altman S (2001). Inhibition of Escherichia coli viability by external guide sequences complementary to two essential genes. Proc Natl Acad Sci USA.

[B10] Li Y, Altman S (1996). Cleavage by RNase P of gene N mRNA reduces bacteriophage lambda burst size. Nucleic Acids Res.

[B11] Guerrier-Takada C, Li Y, Altman S (1995). Artificial regulation of gene expression in Escherichia coli by RNase P. Proc Natl Acad Sci USA.

[B12] Guerrier-Takada C, Salavati R, Altman S (1997). Phenotypic conversion of drug-resistant bacteria to drug sensitivity. Proc Natl Acad Sci USA.

[B13] Yang YH, Li H, Zhou T, Kim K, Liu F (2006). Engineered external guide sequences are highly effective in inducing RNase P for inhibition of gene expression and replication of human cytomegalovirus. Nucleic Acids Res.

[B14] Kovrigina E, Wesolowski D, Altman S (2003). Coordinate inhibition of expression of several genes for protein subunits of human nuclear RNase P. Proc Natl Acad Sci USA.

[B15] Kovrigina E, Yang L, Pfund E, Altman S (2005). Regulated expression of functional external guide sequences in mammalian cells using a U6 RNA polymerase III promoter. RNA.

[B16] Plehn-Dujowich D, Altman S (1998). Effective inhibition of influenza virus production in cultured cells by external guide sequences and ribonuclease P. Proc Natl Acad Sci USA.

[B17] Li H, Trang P, Kim K, Zhou T, Umamoto S, Liu F (2006). Effective inhibition of human cytomegalovirus gene expression and growth by intracellular expression of external guide sequence RNA. RNA.

[B18] Liu F, Altman S (1995). Inhibition of viral gene expression by the catalytic RNA subunit of RNase P from Escherichia coli. Genes Dev.

[B19] Yuan Y, Altman S (1994). Selection of guide sequences that direct efficient cleavage of mRNA by human ribonuclease P. Science.

[B20] Rangarajan S, Raj ML, Hernandez JM, Grotewold E, Gopalan V (2004). RNase P as a tool for disruption of gene expression in maize cells. Biochem J.

[B21] Dykxhoorn DM, Novina CD, Sharp PA (2003). Killing the messenger: short RNAs that silence gene expression. Nat Rev Mol Cell Biol.

[B22] Rossi JJ (1999). Ribozymes, genomics and therapeutics. Chem Biol.

[B23] Stein CA, Cheng YC (1993). Antisense oligonucleotides as therapeutic agents–is the bullet really magical?. Science.

[B24] Santoro SW, Joyce GF (1997). A general purpose RNA-cleaving DNA enzyme. Proc Natl Acad Sci USA.

[B25] Wong-Staal F, Poeschla EM, Looney DJ (1998). A controlled, Phase 1 clinical trial to evaluate the safety and effects in HIV-1 infected humans of autologous lymphocytes transduced with a ribozyme that cleaves HIV-1 RNA. Hum Gene Ther.

[B26] Scherer LJ, Rossi JJ (2003). Approaches for the sequence-specific knockdown of mRNA. Nat Biotechnol.

[B27] Hammond SM, Bernstein E, Beach D, Hannon GJ (2000). An RNA-directed nuclease mediates post-transcriptional gene silencing in Drosophila cells. Nature.

[B28] Guerrier-Takada C, Altman S (2000). Inactivation of gene expression using ribonuclease P and external guide sequences. Methods Enzymol.

[B29] Kim K, Liu F (2007). Inhibition of gene expression in human cells using RNase P-derived ribozymes and external guide sequences. Biochim Biophys Acta.

[B30] Fraser AG, Kamath RS, Zipperlen P, Martinez-Campos M, Sohrmann M, Ahringer J (2000). Functional genomic analysis of C. elegans chromosome I by systematic RNA interference. Nature.

[B31] Gonczy P, Echeverri C, Oegema K, Coulson A, Jones SJ, Copley RR, Duperon J, Oegema J, Brehm M, Cassin E (2000). Functional genomic analysis of cell division in C. elegans using RNAi of genes on chromosome III. Nature.

[B32] Piano F, Schetter AJ, Mangone M, Stein L, Kemphues KJ (2000). RNAi analysis of genes expressed in the ovary of Caenorhabditis elegans. Curr Biol.

[B33] Hanazawa M, Mochii M, Ueno N, Kohara Y, Iino Y (2001). Use of cDNA subtraction and RNA interference screens in combination reveals genes required for germ-line development in Caenorhabditis elegans. Proc Natl Acad Sci USA.

[B34] Maeda I, Kohara Y, Yamamoto M, Sugimoto A (2001). Large-scale analysis of gene function in Caenorhabditis elegans by high-throughput RNAi. Curr Biol.

[B35] Zipperlen P, Fraser AG, Kamath RS, Martinez-Campos M, Ahringer J (2001). Roles for 147 embryonic lethal genes on C.elegans chromosome I identified by RNA interference and video microscopy. EMBO J.

[B36] Ashrafi K, Chang FY, Watts JL, Fraser AG, Kamath RS, Ahringer J, Ruvkun G (2003). Genome-wide RNAi analysis of Caenorhabditis elegans fat regulatory genes. Nature.

[B37] Kamath RS, Fraser AG, Dong Y, Poulin G, Durbin R, Gotta M, Kanapin A, Le BN, Moreno S, Sohrmann M (2003). Systematic functional analysis of the Caenorhabditis elegans genome using RNAi. Nature.

[B38] Lee SS, Lee RY, Fraser AG, Kamath RS, Ahringer J, Ruvkun G (2003). A systematic RNAi screen identifies a critical role for mitochondria in C. elegans longevity. Nat Genet.

[B39] Pothof J, van HG, Thijssen K, Kamath RS, Fraser AG, Ahringer J, Plasterk RH, Tijsterman M (2003). Identification of genes that protect the C. elegans genome against mutations by genome-wide RNAi. Genes Dev.

[B40] Simmer F, Moorman C, Linden AM van der, Kuijk E, Berghe PV van den, Kamath RS, Fraser AG, Ahringer J, Plasterk RH (2003). Genome-wide RNAi of C. elegans using the hypersensitive rrf-3 strain reveals novel gene functions. PLoS Biol.

[B41] Vastenhouw NL, Fischer SE, Robert VJ, Thijssen KL, Fraser AG, Kamath RS, Ahringer J, Plasterk RH (2003). A genome-wide screen identifies 27 genes involved in transposon silencing in C. elegans. Curr Biol.

[B42] Rual JF, Ceron J, Koreth J, Hao T, Nicot AS, Hirozane-Kishikawa T, Vandenhaute J, Orkin SH, Hill DE, van den HS (2004). Toward improving Caenorhabditis elegans phenome mapping with an ORFeome-based RNAi library. Genome Res.

[B43] Dillin A, Hsu AL, rantes-Oliveira N, Lehrer-Graiwer J, Hsin H, Fraser AG, Kamath RS, Ahringer J, Kenyon C (2002). Rates of behavior and aging specified by mitochondrial function during development. Science.

[B44] Piano F, Schetter AJ, Morton DG, Gunsalus KC, Reinke V, Kim SK, Kemphues KJ (2002). Gene clustering based on RNAi phenotypes of ovary-enriched genes in C. elegans. Curr Biol.

[B45] Fire A, Xu S, Montgomery MK, Kostas SA, Driver SE, Mello CC (1998). Potent and specific genetic interference by double-stranded RNA in Caenorhabditis elegans. Nature.

[B46] Zuker M (2003). Mfold web server for nucleic acid folding and hybridization prediction. Nucleic Acids Res.

[B47] McClain WH, Guerrier-Takada C, Altman S (1987). Model substrates for an RNA enzyme. Science.

[B48] Guerrier-Takada C, McClain WH, Altman S (1984). Cleavage of tRNA precursors by the RNA subunit of E. coli ribonuclease P (M1 RNA) is influenced by 3'-proximal CCA in the substrates. Cell.

[B49] Sprinzl M, Dank N, Nock S, Schon A (1991). Compilation of tRNA sequences and sequences of tRNA genes. Nucleic Acids Res.

[B50] Zhu J, Trang P, Kim K, Zhou T, Deng H, Liu F (2004). Effective inhibition of Rta expression and lytic replication of Kaposi's sarcoma-associated herpesvirus by human RNase P. Proc Natl Acad Sci USA.

[B51] Zhou T, Kim J, Kilani AF, Kim K, Dunn W, Jo S, Nepomuceno E, Liu F (2002). In vitro selection of external guide sequences for directing RNase P-mediated inhibition of viral gene expression. J Biol Chem.

[B52] Ma Y, Creanga A, Lum L, Beachy PA (2006). Prevalence of off-target effects in Drosophila RNA interference screens. Nature.

[B53] Kulkarni MM, Booker M, Silver SJ, Friedman A, Hong P, Perrimon N, Mathey-Prevot B (2006). Evidence of off-target effects associated with long dsRNAs in Drosophila melanogaster cell-based assays. Nat Methods.

[B54] Scacheri PC, Rozenblatt-Rosen O, Caplen NJ, Wolfsberg TG, Umayam L, Lee JC, Hughes CM, Shanmugam KS, Bhattacharjee A, Meyerson M (2004). Short interfering RNAs can induce unexpected and divergent changes in the levels of untargeted proteins in mammalian cells. Proc Natl Acad Sci USA.

[B55] Kawa D, Wang J, Yuan Y, Liu F (1998). Inhibition of viral gene expression by human ribonuclease P. RNA.

[B56] Theresa Stiernagle (2005). Theresa Stiernagle. Maintenance of C.elegans. WormBook, ed. The C.elegans Research Community, WormBook, doi/10.1895/wormbook.1.7.1. WormBook.

