# Supplementary figures and images for: Generation of an external guide sequence library for a reverse genetic screen in Caenorhabditis elegans
Source: BMC Biotechnol. 2009 May 20;9:47. doi: 10.1186/1472-6750-9-47 (PMC2696436; doi:10.1186/1472-6750-9-47)

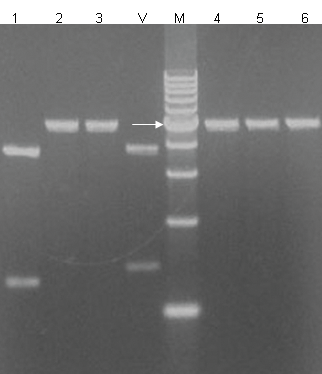

Supplement: Additional File 1 — HincII digestion pattern. The HincII digestion products of the pET28a-EGS clone (lane 1) containing two or three HincII sites, the pET28a-EGS clones (lanes 2–6) containing one HincII site, and pET28a (lane V). The arrow indicates the 5-kb DNA band (lane M). [file 1472-6750-9-47-S1.tiff]

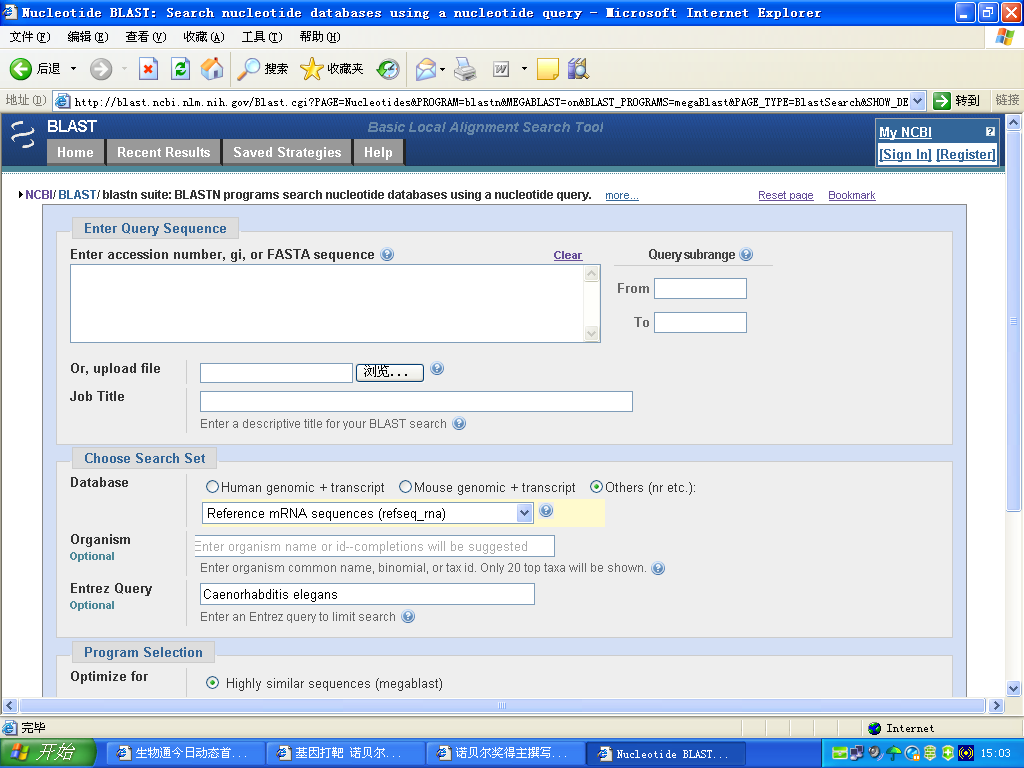

Supplement: Additional File 2 — All candidate target mRNAs of an EGS were identified by BLAST analysis of the target sequence. The NCBI-BLAST web interface. [file 1472-6750-9-47-S2.tiff]

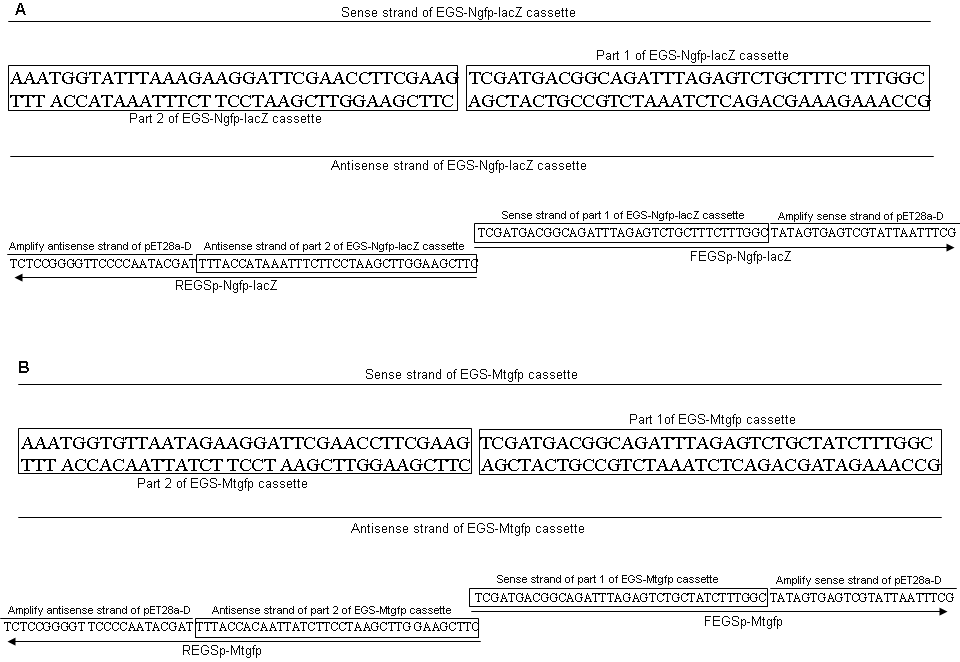

Supplement: Additional File 3 — Demonstration of primer pair. (A) The primer pair of FEGSp-Ngfp-lacZ and REGSp-Ngfp-lacZ. The partially randomized oligonucleotides of FEGSp-Ngfp-lacZ and REGSp-Ngfp-lacZ were composed of two parts. One was used to amplify pET28a-D, which is equal to pET28a but without the fragment between the T7 terminator and T7 promoter. The other was used to amplify the EGS-Ngfp-lacZ cassette. (B) The primer pair of FEGSp-Mtgfp and REGSp-Mtgfp. The partially randomized oligonucleotides of FEGSp-Mtgfp and REGSp-Mtgfp were composed of two parts. One was used to amplify pET28a-D; the other was used to amplify EGS-Mtgfp cassette. [file 1472-6750-9-47-S3.tiff]

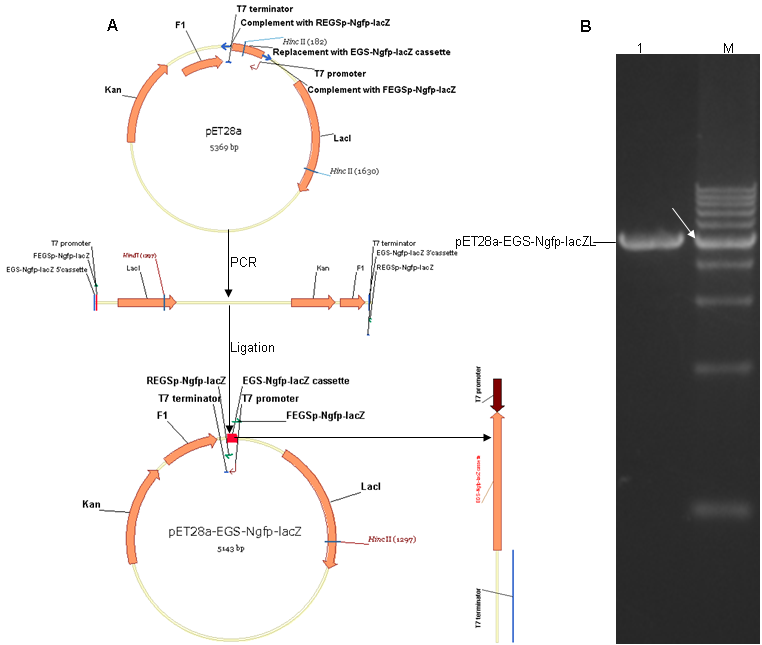

Supplement: Additional File 4 — Construction of pET28a-EGS-Ngfp-lacZ. (A) Flow chart showing construction of pET28a-EGS-Ngfp-lacZ. (B) The PCR product of pET28a-EGS-Ngfp-lacZL (lane 1). The arrow indicates the 5-kb DNA band (lane M). [file 1472-6750-9-47-S4.tiff]

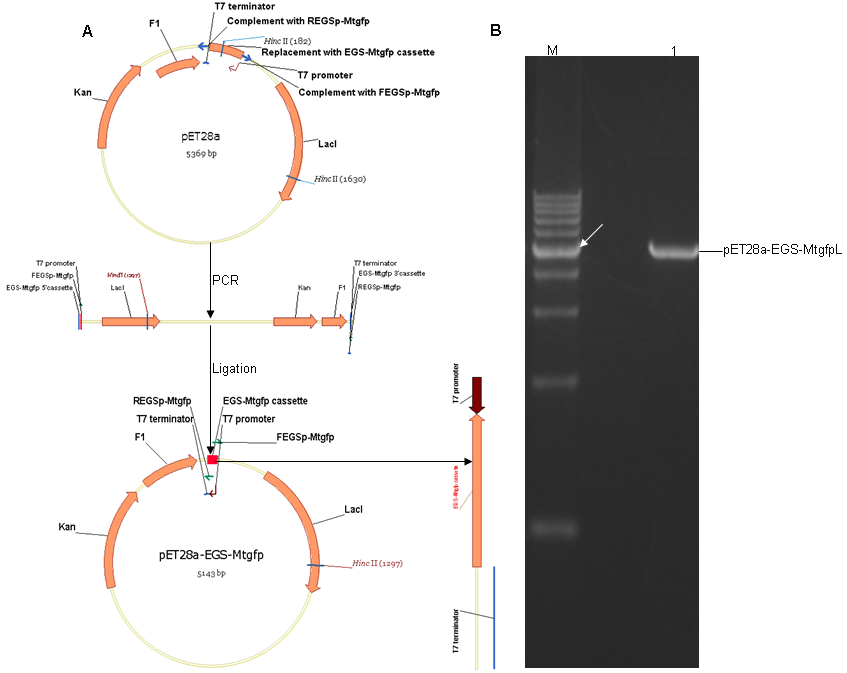

Supplement: Additional File 5 — Construction of pET28a-EGS-Mtgfp. (A) Flow chart showing pET28a-EGS-Mtgfp construction. (B) The PCR product of pET28a-EGS-MtgfpL (lane 1). The arrow indicates the 5-kb DNA band (lane M). [file 1472-6750-9-47-S5.tiff]

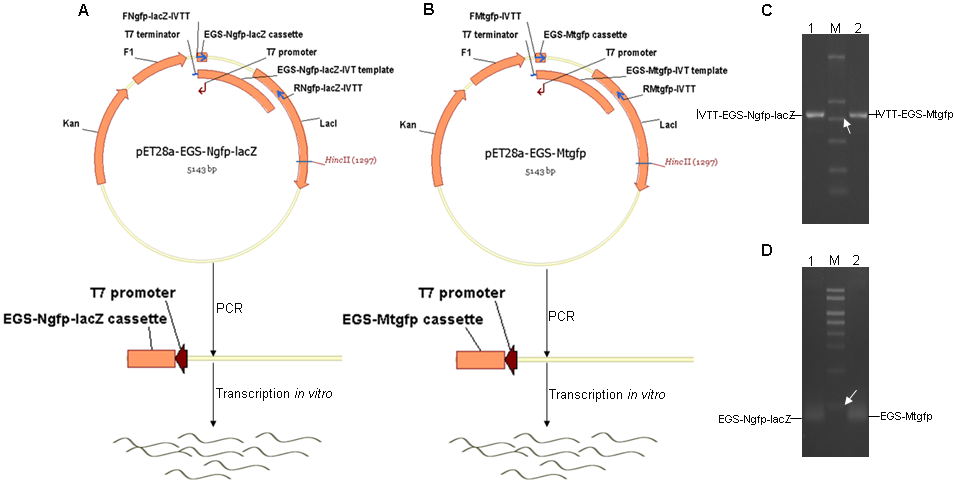

Supplement: Additional File 6 — Preparations of EGS-Ngfp-lacZ and EGS-Mtgfp. (A) Flow chart showing preparation of EGS-Ngfp-lacZ. (B) Flow chart showing preparation of EGS-Mtgfp. (C) The PCR products of IVTT-EGS-Ngfp-lacZ (lane 1) and IVTT-EGS-Mtgfp (lane 2). The arrow indicates the 750-bp DNA band (lane M). (D) The transcription products of EGS-Ngfp-lacZ (lane 1) and EGS-Mtgfp (lane2). The arrow indicates the 100-bp RNA band (lane M). [file 1472-6750-9-47-S6.tiff]

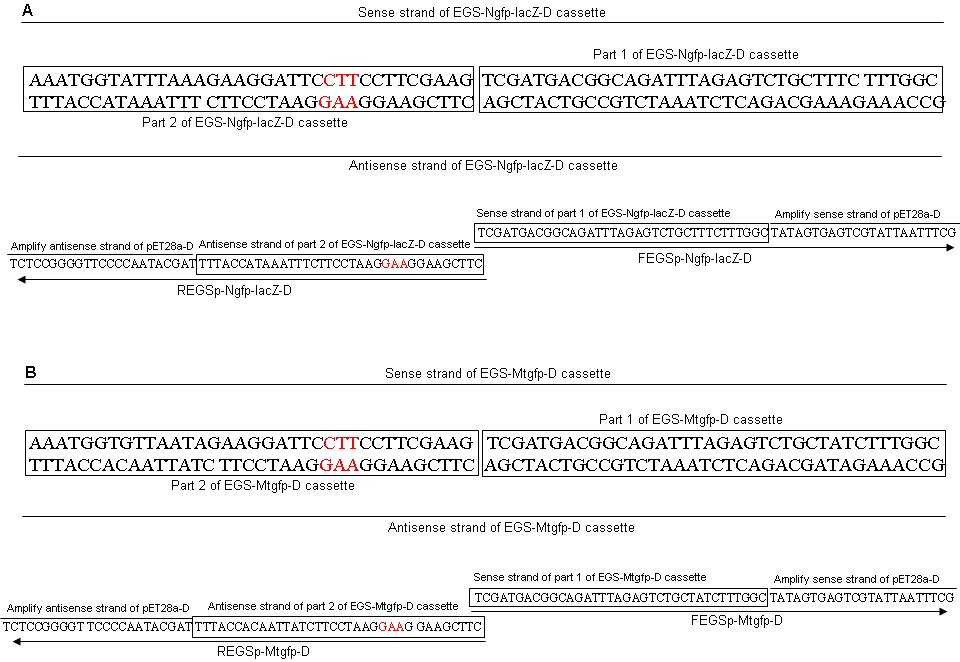

Supplement: Additional File 7 — Demonstration of primer pair. (A) The primer pair of FEGSp-Ngfp-lacZ-D and REGSp-Ngfp-lacZ-D. The partially randomized oligonucleotides of FEGSp-Ngfp-lacZ-D and REGSp-Ngfp-lacZ-D were composed of two parts. One was used to amplify pET28a-D, which is equal to pET28a but without the fragment between the T7 terminator and T7 promoter. The other was used to amplify the EGS-Ngfp-lacZ-D cassette. (B) The primer pair of FEGSp-Mtgfp-D and REGSp-Mtgfp-D. The partially randomized oligonucleotides of FEGSp-Mtgfp-D and REGSp-Mtgfp-D were composed of two parts. One was used to amplify pET28a-D; the other was used to amplify EGS-Mtgfp-D cassette. [file 1472-6750-9-47-S7.tiff]

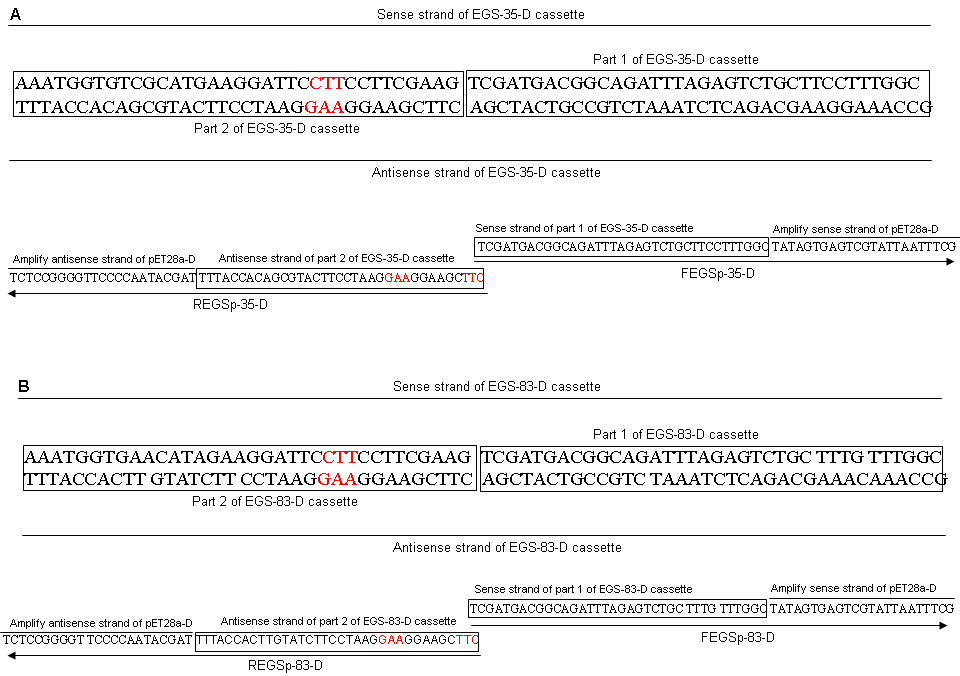

Supplement: Additional File 8 — Demonstration of primer pair. (A) The primer pair of FEGSp-35-D and REGSp-35-D. The partially randomized oligonucleotides of FEGSp-35-D and REGSp-35-D were composed of two parts. One was used to amplify pET28a-D, which is equal to pET28a but without the fragment between the T7 terminator and T7 promoter. The other was used to amplify the EGS-35-D cassette. (B) The primer pair of FEGSp-83-D and REGSp-83-D. The partially randomized oligonucleotides of FEGSp-83-D and REGSp-83-D were composed of two parts. One was used to amplify pET28a-D; the other was used to amplify the EGS-83-D cassette. [file 1472-6750-9-47-S8.tiff]

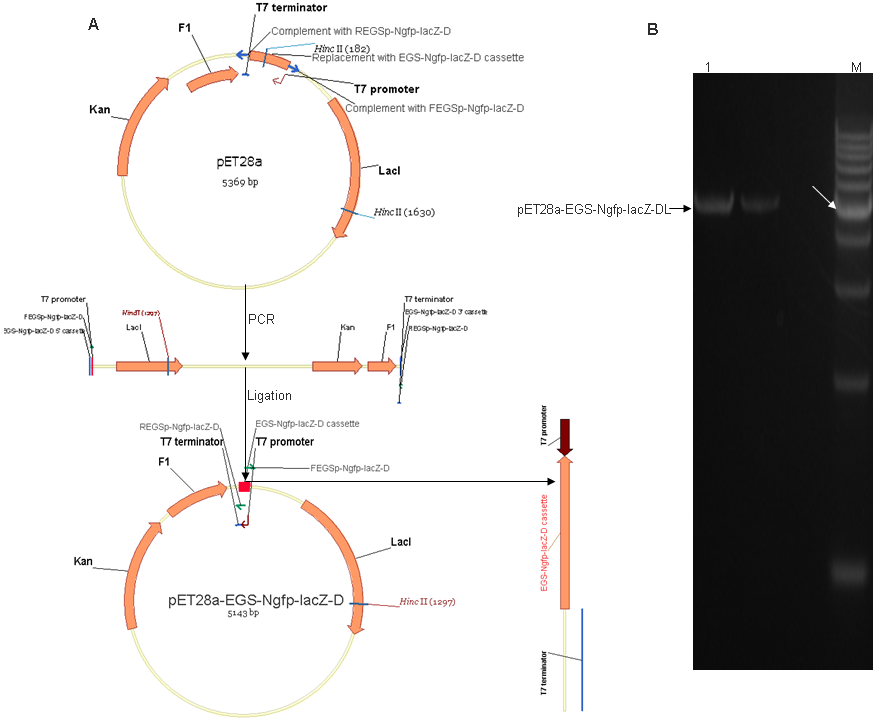

Supplement: Additional File 9 — Construction of pET28a-EGS-Ngfp-lacZ-D. (A) Flow chart showing construction of pET28a-EGS-Ngfp-lacZ-D. (B) The PCR product of pET28a-EGS-Ngfp-lacZ-DL (lane 1). The arrow indicates the 5-kb DNA band (lane M). [file 1472-6750-9-47-S9.tiff]

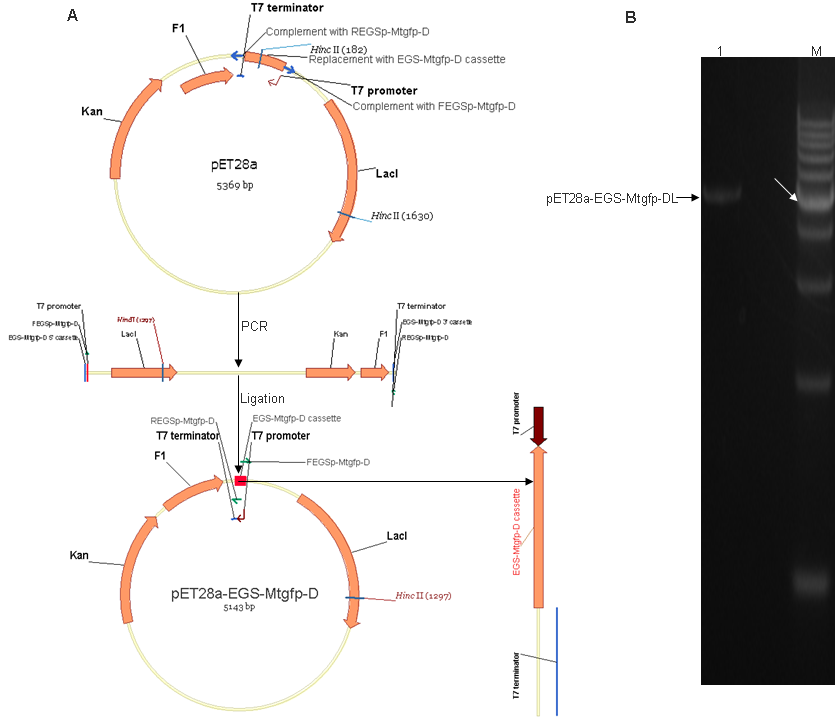

Supplement: Additional File 10 — Construction of pET28a-EGS-Mtgfp-D. (A) Flow chart showing construction pET28a-EGS-Mtgfp-D. (B) The PCR product of pET28a-EGS-Mtgfp-DL (lane 1). The arrow indicates the 5-kb DNA band (lane M). [file 1472-6750-9-47-S10.tiff]

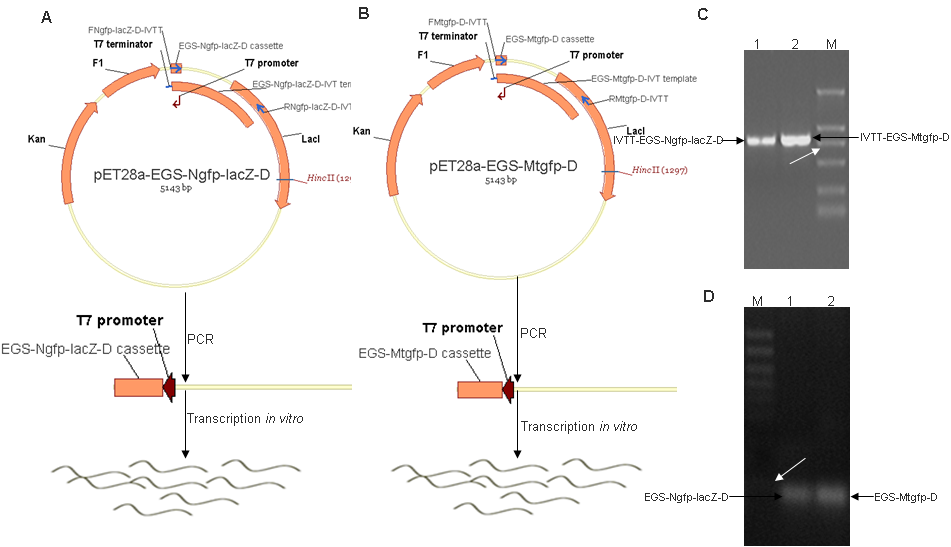

Supplement: Additional File 11 — Preparations of EGS-Ngfp-lacZ-D and EGS-Mtgfp-D. (A) Flow chart showing preparation of EGS-Ngfp-lacZ-D. (B) Flow chart showing preparation of EGS-Mtgfp-D. (C) The PCR products of IVTT-EGS-Ngfp-lacZ-D (lane 1) and IVTT-EGS-Mtgfp-D (lane 2). The arrow indicates the 750-bp DNA band (lane M). (D) The transcription products of EGS-Ngfp-lacZ-D (lane 1) and EGS-Mtgfp-D (lane2). The arrow indicates the 100-bp RNA band (lane M). [file 1472-6750-9-47-S11.tiff]

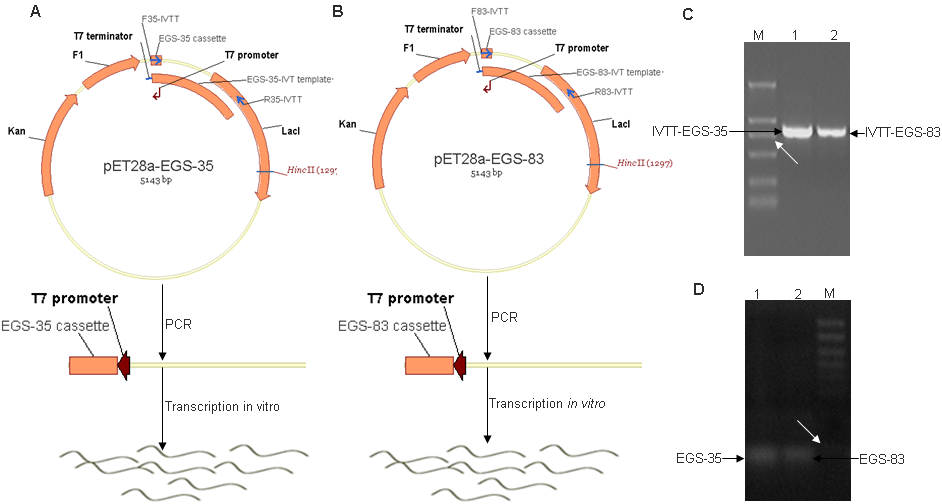

Supplement: Additional File 12 — Preparations of EGS-35 and EGS-83. (A) Flow chart showing preparation of EGS-35. (B) Flow chart showing preparation of EGS-83. (C) The PCR products of IVTT-EGS-35 (lane 1) and IVTT-83 (lane 2). The arrow indicates the 750-bp DNA band (lane M). (D) The transcription products of EGS-35 (lane 1) and EGS-83 (lane 2). The arrow indicates the 100-bp RNA band (lane M). [file 1472-6750-9-47-S12.tiff]

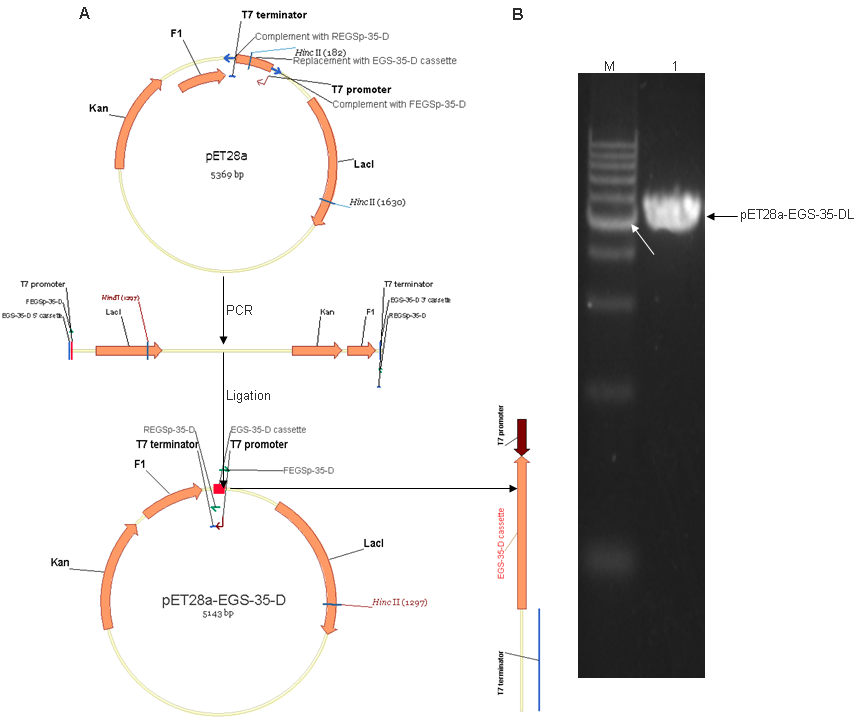

Supplement: Additional File 13 — Construction of pET28a-EGS-35-D. (A) Flow chart showing construction of pET28a-EGS-35-D. (B) The PCR product of pET28a-EGS-35-DL (lane 1). The arrow indicates the 5-kb DNA band (lane M). [file 1472-6750-9-47-S13.tiff]

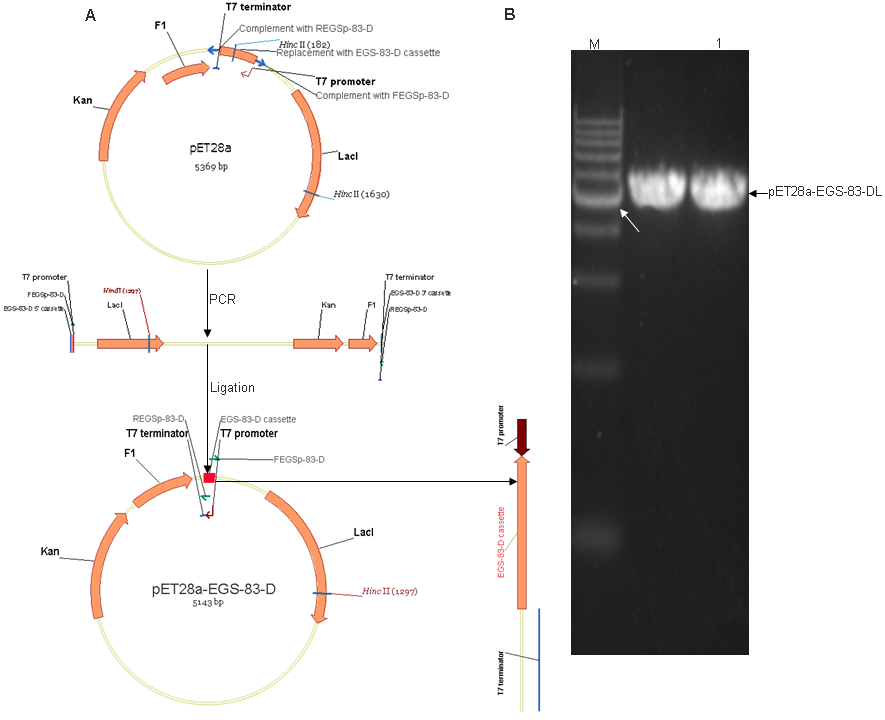

Supplement: Additional File 14 — Construction of pET28a-EGS-83-D. (A) Flow chart showing construction of pET28a-EGS-83-D. (B) The PCR product of pET28a-EGS-83-DL (lane 1). The arrow indicates the 5-kb DNA band (lane M). [file 1472-6750-9-47-S14.tiff]

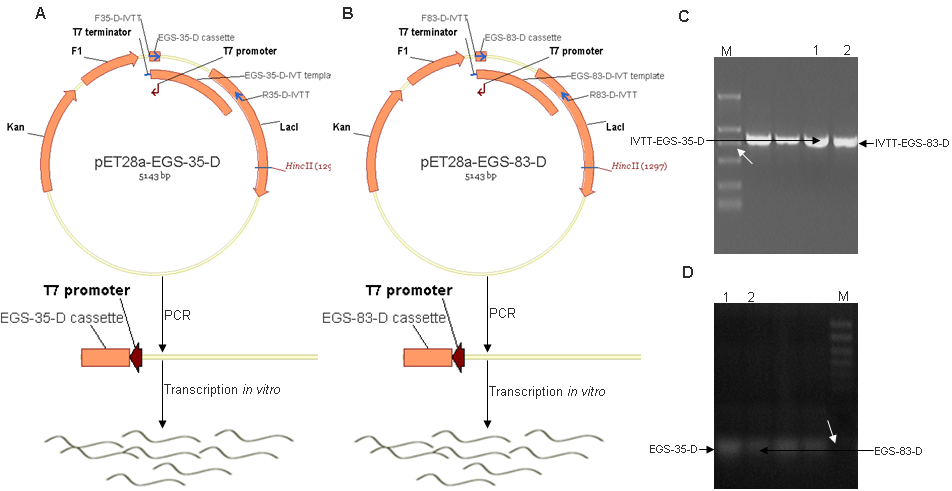

Supplement: Additional File 15 — Preparations of EGS-35-D and EGS-83-D. (A) Flow chart showing preparation of EGS-35Z-D. (B) Flow chart showing preparation of EGS-83-D. (C) The PCR products of IVTT-EGS-35-D (lane 1) and IVTT-EGS-83-D (lane 2). The arrow indicates the 750-bp DNA band (lane M). (D) The transcription products of EGS-35-D (lane 1) and EGS-83-D (lane 2). The arrow indicates the 100-bp RNA band (lane M). [file 1472-6750-9-47-S15.tiff]
